# Structural and mechanistic basis of the central energy-converting methyltransferase complex of methanogenesis

**DOI:** 10.1073/pnas.2315568121

**Published:** 2024-03-26

**Authors:** Iram Aziz, Kanwal Kayastha, Susann Kaltwasser, Janet Vonck, Sonja Welsch, Bonnie J. Murphy, Jörg Kahnt, Di Wu, Tristan Wagner, Seigo Shima, Ulrich Ermler

**Affiliations:** ^a^Molecular Membrane Biology, Max Planck Institute of Biophysics, Frankfurt am Main D-60438, Germany; ^b^Central Electron Microscopy Facility, Max Planck Institute of Biophysics, Frankfurt am Main D-60438, Germany; ^c^Structural Biology, Max Planck Institute of Biophysics, Frankfurt am Main D-60438, Germany; ^d^Redox and Metalloprotein Research Group, Max Planck Institute of Biophysics, Frankfurt am Main D-60438, Germany; ^e^Max Planck Institute for Terrestrial Microbiology, Marburg D-35043, Germany; ^f^Max Planck Institute for Marine Microbiology, Bremen D-28359, Germany

**Keywords:** methanogenesis, sodium-ion translocation, methyltransferase, vitamin B_12_, cryo-EM

## Abstract

Methanogenic archaea inhabiting anaerobic environments play a crucial role in the global biogeochemical material cycle. The most universal electrogenic reaction of their methane-producing energy metabolism is catalyzed by *N    *^5^-methyl-tetrahydromethanopterin: coenzyme M methyltransferase (MtrABCDEFGH), which couples the vectorial Na^+^ transport with a methyl transfer between the one-carbon carriers tetrahydromethanopterin and coenzyme M via a vitamin B_12_ derivative (cobamide) as prosthetic group. We present the 2.08 Å cryo-EM structure of Mtr(ABCDEFG)_3_ composed of the central Mtr(ABFG)_3_ stalk symmetrically flanked by three membrane-spanning MtrCDE globes. Tetraether glycolipids visible in the map fill gaps inside the multisubunit complex. Putative coenzyme M and Na^+^ were identified inside or in a side-pocket of a cytoplasmic cavity formed within MtrCDE. Its bottom marks the gate of the transmembrane pore occluded in the cryo-EM map. By integrating Alphafold2 information, functionally competent MtrA–MtrH and MtrA–MtrCDE subcomplexes could be modeled and thus the methyl-tetrahydromethanopterin demethylation and coenzyme M methylation half-reactions structurally described. Methyl-transfer-driven Na^+^ transport is proposed to be based on a strong and weak complex between MtrCDE and MtrA carrying vitamin B_12_, the latter being placed at the entrance of the cytoplasmic MtrCDE cavity. Hypothetically, strongly attached methyl-cob(III)amide (His-on) carrying MtrA induces an inward-facing conformation, Na^+^ flux into the membrane protein center and finally coenzyme M methylation while the generated loosely attached (or detached) MtrA carrying cob(I)amide (His-off) induces an outward-facing conformation and an extracellular Na^+^ outflux. Methyl-cob(III)amide (His-on) is regenerated in the distant active site of the methyl-tetrahydromethanopterin binding MtrH implicating a large-scale shuttling movement of the vitamin B_12_-carrying domain.

## Significance

Annually, 1 to 2 Gt of the potent greenhouse gas and biofuel methane are produced by methanogenic archaea. A key enzyme of their energy metabolism is a Na^+^ pumping and vitamin B_12_-dependent methyltransferase. Here, we present a cryo-EM structure of this multisubunit membrane protein complex that provides the structural information about its unusual architecture and the unique coupling principle between a chemical and an ion-gradient forming process via a vitamin B_12_ derivative as motor. The energy is provided by two exergonic methyl transfer reactions producing two different Co oxidation and ligation states. It is postulated that the methyl-Co(III) (His-on) state creates an intracellular (inward) entrance and the Co(I) (His-off) state an extracellular (outward) exit for transmembrane Na^+^ translocation.

Anaerobic biomass degradation, a vital process of the global carbon cycle, includes the degradation of 1 to 2% of the photosynthetically produced organic matter to methane (ca. 1 to 2 Gt per year) (1). The terminal methane-forming step is executed by a specialized group of archaea, the methanogens (2, 3), which use a variety of substrates as CO_2_ with H_2_, methanol, and other C_1_ compounds, acetate and methoxylated aromatic compounds (as well as several others) (4, 5). Their conversions to methane slightly differ, although, in principle, common biochemical reactions and enzyme pools are employed. The biochemically characterized hydrogenotrophic, methylotrophic, acetoclastic, and methoxy-compounds utilizing variants share coenzymes methanofuran (MFR), tetrahydromethanopterin (H_4_MPT), and coenzyme M (CoM) as carriers for one-carbon compounds at different oxidation states (*SI Appendix*, Fig. S1) (6–8). The central electrogenic reaction of most methanogenic variants is the exergonic methyl transfer from methyl-H_4_MPT to CoM (ΔG°' = −30 kJ/mol) coupled with a vectorial Na^+^ transport from the cytoplasm to the cell exterior (9). In methylotrophic archaea that disproportionate methanol to methane and CO_2_, this reaction runs backward and the chemiosmotic energy of the Na^+^ gradient drives endergonic methyl transfer from methyl-CoM to H_4_MPT. Anaerobic oxidation of methane to CO_2_ performed by methanotrophic archaea largely uses the same catalytic machinery but completely proceeds in the reverse direction (10, 11).

N*^5^*-methyl-H_4_MPT:CoM methyltransferase, catalyzing the methyltransferase reaction, was comprehensively studied since ca. 1990 predominantly from enzymes isolated from *Methanothermobacter marburgensis* (12–16) and *Methanosarcina mazei* (17–19). It was established as a membrane protein complex of eight different subunits (MtrABCDEFGH) that couples methyl transfer with Na^+^ pumping. While MtrCDE are integral membrane subunits, MtrABFG only possess one membrane-spanning helix each as a membrane anchor. MtrA, in addition, contains a soluble domain carrying 5-hydroxybenzimidazolyl cobamide, a vitamin B_12_ (cobalamin) derivative (20, 21). Soluble MtrH, hosting the binding site for methyl-H_4_MPT is cytoplasmically attached to the residual MtrABCDEFG complex and easily lost during preparation (22). The MtrABCDEFG complex devoid of MtrH was characterized as a trimer with a molecular mass of 430 kDa (23). Methyl transfer is mediated in a two-step process via the Co-containing vitamin B_12_ derivative. The first half reaction consists of a methyl transfer from CH_3_-H_4_MPT to cob(I)amide [I] and the second half reaction from CH_3_-cob(III)amide to CoM [II] (12, 14). In the two nucleophilic substitution reactions, the oxidation state formally changes from Co(I) to Co(III) and back from Co(III) to Co(I).

[I] $$\begin{array}{c} {\text{CH}}_{3}\;-\;H_{4}\text{MPT}\;+\;\text{cob}\left(I\right)\text{amide}\;H_{4}\text{MPT}+\;{\mathrm{CH}}_{3}\;-\;\text{cob}\left(\text{III}\right)\text{amide}, \end{array}$$

[II] $$\begin{array}{c} \text{CoM}+{\text{CH}}_{3}-\text{cob}\left(\text{III}\right)\text{amide}{\text{CH}}_{3}-\text{CoM}+\text{cob}\left(I\right)\text{amide}. \end{array}$$

Only the exergonic demethylation of CH_3_-cob(III)amide is Na^+^-dependent and most likely powers the endergonic ion gradient formation (14, 15, 17). Based on the measured ^22^Na^+^ content in ether lipid liposomes reconstituted with purified MtrABCDEDFGH of *M. mazei* (18) about two Na^+^ ions cross the membrane per methyl group transferred.

The mode of action of this unique ion pump, an example of ancient Na^+^ bioenergetics (24), is completely unknown on an atomic level because no structural information is available yet. In this report, we present the single-particle cryogenic electron microscopy (cryo-EM) structure of a subcomplex without and with CoM at 2.08 Å and 2.39 Å resolution, respectively. We complement these data with Alphafold2 models for MtrH and the soluble MtrA domain, which allows us to construct a first approximate picture of the entire methyltransferase coupled Na^+^ transport.

## Results and Discussion

### Structure Determination and Overall Architecture of the Mtr(ABCDEFG)_3_ Complex.

**Fig. 1.**
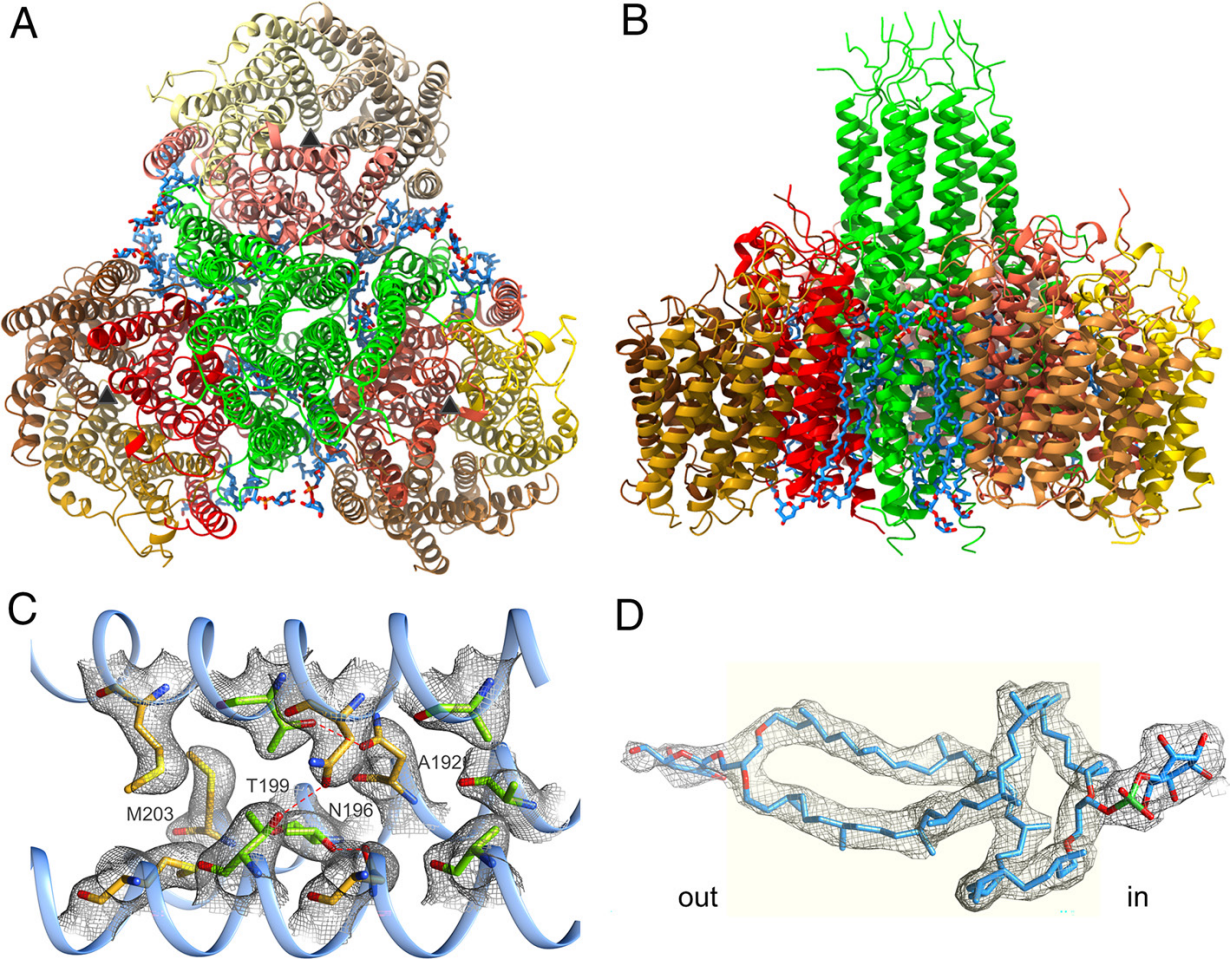
Overall architecture of the Mtr(ABCDEFG)_3_ complex of *M. marburgensis*. (*A*) Model viewed parallel (from the cytoplasmic side) and (*B*) perpendicular to the membrane monolayer. The multisubunit complex is composed of three MtrCDE globes (MtrC brown, MtrD yellow, and MtrE red) attached to the membrane section of the central Mtr(A_c_BFG)_3_ stalk (green). No contact area is formed between the three MtrCDE globes. The pseudo-threefold axes, marked by black triangles, indicate the location of the MtrCDE cavity and the locked membrane pore deeper inside. Firmly bound methanogenic tetraether glycolipids are shown as sticks (carbon in blue). (*C*) Section of the three-helix bundle of MtrA_c_ at the threefold axis. Three identical, predominantly hydrophobic side chains are arranged as layers perpendicular to the helix axes and form the core of the stalk. In the 1. and 3. layer, the carbons are shown in gold, in the 2. and 4. layer in light green. The cryo-EM density is drawn in gray. (*D*) The tetraether glycolipid. The density (gray) was interpreted as gentiobiosyl GDPT-phosphoinositol, the most abundant tetraether lipid in *M. marburgensis* (28). The lipid crosses the entire cell membrane (light yellow). The density of the terminal glycoside at the extracellular side is highly blurred.

The weak association of MtrH to the MtrABCDEFG core complex of *M. marburgensis* (22) prompted us to initially produce a highly homogeneous enzyme by treating the enzyme with dimethyl maleic anhydride for complete removal of MtrH (23). The cryo-EM structure could be determined at 2.37 Å resolution using Relion (25) (*SI Appendix*, Fig. S2). As the purification of Mtr from *Methanothermobacter wolfeii* appeared to give rise to a protein complex containing MtrH, a cryo-EM structure was determined at 3.3 Å resolution (*SI Appendix*, Fig. S3). In parallel, the *M. marburgensis* cells were gently disrupted with pseudomurein endopeptidase to prevent loss of MtrH (26). Although sodium dodecyl sulfate–polyacrylamide gel electrophoresis (SDS-PAGE) and mass-spectrometric data indicated that MtrH was at least partly bound to the *M. marburgensis* and *M. wolfeii* enzymes after purification (*SI Appendix*, Figs. S3 and S4), cryo-EM maps determined from these samples were virtually identical with no additional density corresponding to MtrH. It is unclear from this result, whether MtrH is in a disordered state or has dissociated during cryo-EM grid preparation. Therefore, the structural analysis is based on a 2.08 Å resolution map of *M. marburgensis* [reconstituted with Relion (25), Table 1 and *SI Appendix*, Fig. S5, EMD-18135 that reveals the enzyme as trimer of the protomer MtrABCDEFG (Fig. 1 *A* and *B*) in line with previous light-scattering and mass-spectrometric data (23). The resolution ranges between 1.9 Å in the core and 2.5 Å in the periphery (*SI Appendix*, Fig. S5*C*). The excellent quality of the map is displayed in Fig. 1 *C* and *D*. After density modification with Phenix (27) the estimated resolution was improved to 1.99 Å. Model building was possible for most of MtrB (B31-B100; total residues 100), MtrC (C12-C264; 267), MtrD (D1-D233; 233), MtrE (E1-E294; 295), MtrF (F1-F68; 68), and MtrG (G8-G81; 86). For MtrA, only the C-terminal anchor helix MtrA_c_ (178:238) was visible but not the soluble MtrA domain (MtrA_s_) carrying the B_12_ derivative. The pink color of the protein solution and protein analytical data (*SI Appendix*, Figs. S2–S4) indicates that MtrA_s_ is not cleaved but structurally disordered. In several cryo-EM maps, weak density was visible parallel to the stalk, which may reflect the presence of flexible MtrA_s_ (*SI Appendix*, Fig. S2*C*).

**Table 1.** Cryo-EM data collection, refinement, and validation statistics

	MtrABCDEFG	MtrABCDEFG soaked with CoM
Deposited files	EMDB-18135 PDB 8Q3V	EMDB-18162 PDB 8Q54
Data collection and processing		
Microscope	FEI Titan Krios G3i	FEI Titan Krios G3i
Voltage (kV)	300	300
Camera	Gatan K3 summit	Gatan K3 summit
Exposure time (s)	3.5	3.0
Total dose (e^−^/Å^2^)	73.9	64.2
Dose per frame (e^−^/Å^2^)	1.85	1.1
Defocus range (μm)	1.2 to 2.1	1.2 to 2.1
Pizel size (Å) (calibrated)	0.837	0.837
Magnification (nominal)	105,000×	105,000×
No. of micrographs	8,210	4,443
Initial particle number	2,783,721	910,067
Final particle number	138,464	87,173
Symmetry applied	C3	C3
Map resolution (Å)	2.08	2.39
FSC threshold Fourier shell correlation	0.143	0.143
Refinement and validation		
Map-sharpening B factor (Å^2^)	−43.8	−53.5
Model composition		
Chains	27	27
Protein (Residues)	3,161	3,156
Ligands (Na^+^, Mg^2+^, CoM, GT)	6, 3, 0, 12	6, 3, 3, 12
Water	208	88
RMSD bond length (Å)	0.006	0.014
RMSD bond angles (°)	0.97	1.44
MolProbity score	1.35	2.1
Clash score	3.7	7.1
Ramachandran plot (%)		
Favored	98.5	97.9
Allowed	1.50	2.1
Outliers	0.0	0.0
Rotamer outliers (%)	1.5	6.9
ADP (B-factor) (min/max/mean)		
Protein	14.5, 136.2, 52.4	30.0, 172.3, 79.2
Ligand	30.0, 74.4, 64.5	62.0, 5435.0, 237.9
Water	30.0, 50.7, 30.8	51.8, 99.7, 74.2

**Fig. 2.**
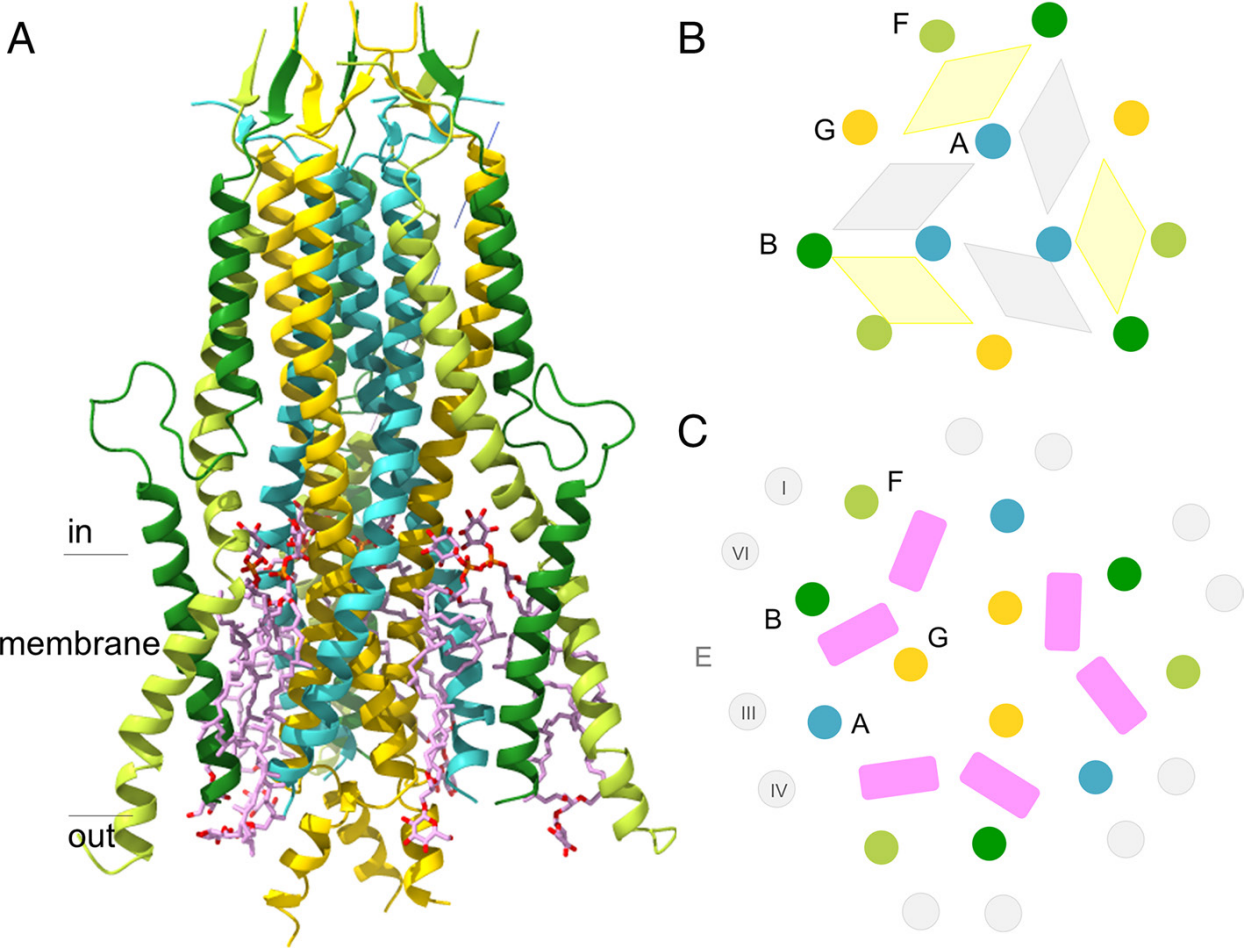
The MtrA_c_BFG stalk. (*A*) Subunit arrangement viewed perpendicular to the membrane monolayer. MtrA_c_ (turquoise), MtrG (yellow), MtrB (green), and MtrF (yellow-green) assemble to a trapezoid-like scaffold referred to as the stalk. Its dimensions are The complex is ca. 105 Å high and the cytoplasmic domain has a diameter of ca. 45 Å (*Top*) and the membrane domain ca. 55 Å (*Bottom*). The N-termini of the four subunits are localized at their cytoplasmic ends and, correspondingly, their C-termini at the extracellular end as predicted previously on the basis of the positive inside rule (9). The helix segment G73:G83 of MtrG significantly extends into the extracellular space after a kink near the membrane boundary. In the cytoplasm, the MtrB helix runs nearly parallel to MtrA_c_, while the MtrF and MtrG helices are slightly inclined outward and inward, respectively. The tetraether lipids are drawn as sticks (carbon in pink). (*B*) Scheme of the intracellular section of the stalk visualized from the membrane side. Helices are drawn as circles filled with colors of (*A*). The four-helix bundles formed by MtrA_c_BFG and MtrA_c_A_c_’BG are characterized by hydrophobic (yellow) and hydrophilic (gray) interiors, respectively. The surface of the cytoplasmic segment of the stalk is dominated by acidic and basic amino acids. (*C*) Scheme of the membrane section of the stalk. Helices III (E86:E117) and IV (E166:E183) of MtrE contact MtrA_c_; helices I (E2:E23) and VI (E234:E257) of MtrE contact MtrB and MtrF. The closely associated MtrAG and MtrBF couples are separated from each other by two tetraether lipids (pink bars).

Architecturally, the trimeric multisubunit membrane complex (PDB code: 8Q3V) is built up of a central stalk composed of a MtrA_c_BFG trimer to whose membrane surface three globular MtrCDE subcomplexes are attached according to the threefold symmetry (Fig. 1 *A* and *B*). The stalk is composed of membrane (45 Å in length), cytoplasmic (45 Å) as well as extracellular sections (15 Å) (Fig. 2*A*). Each membrane spanning MtrCDE globe has a diameter of 50 to 60 Å and forms an interface with all subunits of the MtrA_c_BFG stalk mainly via MtrE (Fig. 2*C*), while MtrC and MtrD are localized at the periphery of the protein complex. Twelve partially disordered tetraether glycolipids (*SI Appendix*, Fig. S6) are firmly attached inside gaps of the protein scaffold thereby acting as stabilizers (*SI Appendix*, Fig. S6*B*). The modeled gentiobiosyl glycerol diacyl glycerol tetraether (GDPT)-phosphoinositol molecules (28, 29) cross the entire monolayer and are oriented with the diglycosidic head towards the extracellular space and the phosphoinositol head toward the cytoplasm (Fig. 1*D* and *SI Appendix*, Fig. S6*A*), the latter being stabilized by specific hydrogen bonds (*SI Appendix*, Fig. S6*C*).

### The Stalk.

MtrA_c_, MtrB, MtrF, and MtrG of the stalk consist of one long, partly interrupted helix and an N-terminal short linear segment pointing into the cytoplasm‚ thereby forming short β-sheet units between MtrB and MtrG as well as MtrA and MtrF (Fig. 2*A*). MtrB contains an additional N-terminal arm of 30 amino acids that are disordered and not visible in the density map. The cytoplasmic section of the stalk contains in its core three straight helices of MtrA_c_ contacting each other along the threefold axis‚ mainly via hydrophobic layers formed by three copies of ProA185, AlaA188, AlaA192, AsnA196, ThrA199, and MetA203, respectively (Fig. 1*C*). Helices of MtrB, MtrF, and MtrG completely encircle the MtrA_c_ trimer such that two different types of four-helix bundles, MtrA_c_BFG and MtrA_c_A_c_’BG, are formed (Fig. 2*B*).

Inside the membrane the three MtrA_c_ helices become bent outwards by ca. 15° (without disturbing the hydrogen bond pattern of the helices) and, in parallel, the inward inclined MtrG helices become straight at the highly conserved GlyG49 (*SI Appendix*, Fig. S7*G*) and form a three-helix bundle (G50:G65; helix definition via starting and ending residues) along the threefold axis (Fig. 2 *A* and *C*). The strictly hydrophobic triangular layers of MtrG at the threefold axis include IleG48, IleG52, LeuG55, ValG59, LeuG62, LeuG71, PheG72, and LeuG75 flanked by further nonpolar residues. At the membrane boundary, the cytoplasmic (37:56) and membrane (74:100) sections of the MtrB helix are displaced by an outward-directed hairpin-shaped linker (Fig. 2*A*). In the membrane, the MtrB helix is inclined ca. 20° toward the threefold axis. The MtrF helix is first kinked toward the threefold axis by ca. 15° at GlyF28 close to the cytoplasmic membrane boundary and toward the opposite direction by ca. 25° at the highly conserved GlyF44 deeply inside the membrane (*SI Appendix*, Fig. S7*F*). While MtrB and MtrF mostly contact each other along the entire stalk, they become separated from the MtrAG couple inside the membrane. The intermediate space is occupied by two tetraether glycolipids (Fig. 2*C* and *SI Appendix*, Fig. S6).

### The MtrCDE Globe.

**Fig. 3.**
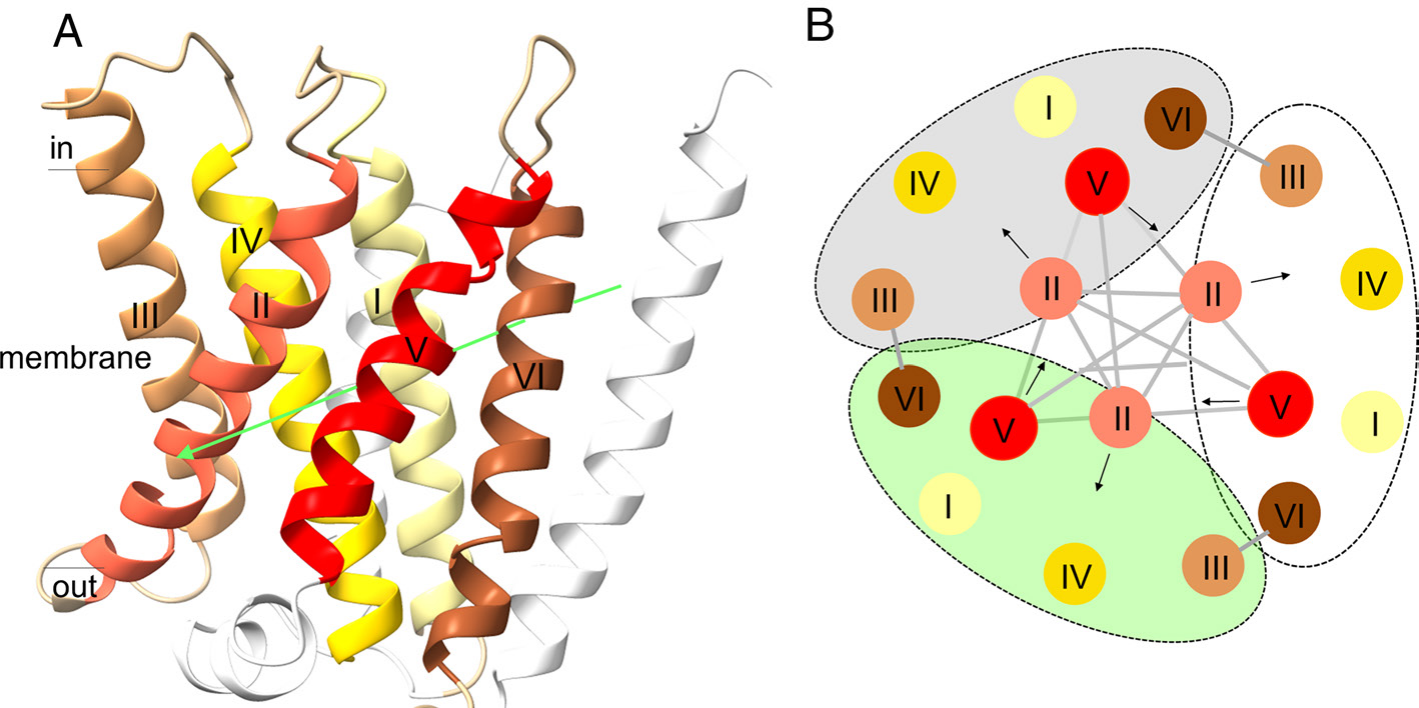
Structure of the six-helix bundle of MtrC, MtrD, and MtrE. (*A*) The MtrC structure. Helices I and IV as well as helices II and V, tilted against each other by ca. 50°, face each other at the longer edge, while helices III and VI occupy the flattened edges in the distorted hexagon-like arrangement. The additional N- and C-terminal helices are shown in light-gray. Like MtrC, MtrD and MtrE contain a six-helix bundle as central membrane-spanning folding unit. The space inside is essentially occupied by hydrophobic side chains. The six-helix bundle begins and ends on the extracellular side as predicted by hydropathy plots and the positive-inside rule (9, 33). Helices I, II, and III and helices IV, V, and VI are related by a pseudo-twofold axis (drawn as green arrow). An inverted topology between groups of helices is frequently found in membrane proteins (34). (*B*) Scheme of the six-helix bundle topology in MtrC, MtrD, and MtrE (lined in gray, white, and green) viewed along the pseudo-threefold axis and from the cytoplasmic side. Helices II coincide at the intracellular membrane boundary and are tilted outwards inside the membrane, while helices V are oriented in the opposite manner (indicated by black arrows) and meet each other at the extracellular membrane boundary. Helices I, III, IV, and VI of MtrC, MtrD and MtrE shield the central helices II and V. An interface is formed between the peripheric helices III and VI and between the central helices II and II, II and V as well as V and V. Only, the six-helix bundle of MtrE interact with the stalk via helices I, III, IV, and VI.

The integral membrane subunits MtrC, MtrD, and MtrE have a similar overall architecture despite an insignificant sequence identity of 8 to 11%. Pairwise structural alignments give rmsd values of 4.4 Å (MtrC/MtrE; 204 of 254 residues used for calculation; TM-score: 0.57), 3.8 Å (MtrD/MtrE; 205 of 233; TM: 0.6), and 3.6 Å (MtrC/MtrD; 181 of 233; TM: 0.59) (30). Six helices (I–VI) form a flattened and distorted hexagon-like shape (Fig. 3*A*), which exhibits only moderate topological similarities to other membrane protein scaffolds according to Dali (31) with the possible exception of the manganese ABC transporter (32). By rotating around a pseudo-twofold axis, helices I–III and IV–VI can be superimposed (Fig. 3*A*); their rms deviation for MtrC is 3.0 Å (55 of 61; TM: 0.49). Unexpectedly, MtrC, MtrD, and MtrE are related to each other by a pseudo-threefold symmetry (Figs. 1*A* and 3*B*). Helices II and V of each six-helix bundle form an inner circle and the other 12 helices an outer circle. Inter-subunit contacts between the helices are marked in Fig. 3*B*. Although they most likely arose from a common ancestor MtrC, MtrD, and MtrE also substantially differ from each other in the number of extra membrane-spanning helices and in the structure of the cytoplasmic regions (*SI Appendix*, Fig. S8) reflecting their specialized roles in the intersubunit assembly (*SI Appendix*, Fig. S9) and function.

### Na^+^ and CoM Binding.

**Fig. 4.**
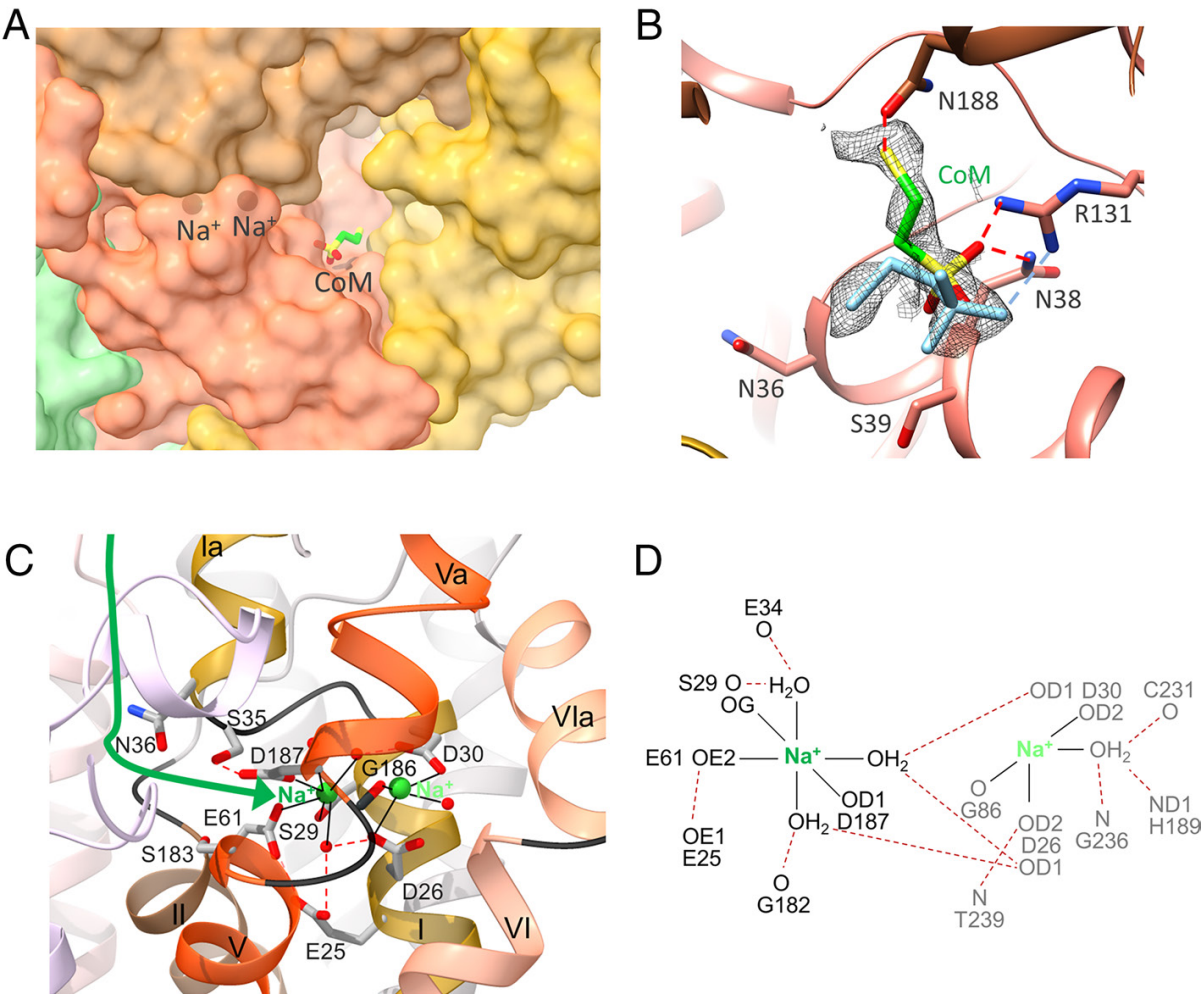
CoM and Na^+^ binding. (*A*) The MtrCDE cavity viewed from the cytoplasmic side. MtrCDE (gold, brown, red) are painted in a transparent surface representation. Putative CoM is attached to the wall and two putative Na^+^ are buried inside a side pocket of the MtrCDE cavity. (*B*) Binding of CoM. CoM is localized in an extra density (gray) of a cryo-EM map of MtrABCDEFG incubated with CoM. In the more highly occupied conformation (carbon in green) the thiol sulfur interacts with AsnC188 while in the less-occupied conformation (in light blue) the thiol points into the cavity. (*C*) Structure of the Na^+^ binding pocket localized in MtrE. The Na^+^ binding pocket is formed at the N-terminal end of helices II and VI and the C-terminal end of helices I and V. The C-terminal end of helix I (E3:E31, gold) ends at GlyE32 and after a lateral linker (black) a short, partially distorted helix Ia (E36:E44) continues into the cytoplasm while the N-terminal end of helix II (E61:E82, brown) ends at the first Na^+^ binding site. At its C-terminal end, helix V (E167:E182, orange-red) is prolonged (helix Va, E187:E193) into the cytoplasm after a kink (black) at the highly conserved GlyE182 and GlyE186. The N-terminal end of helix VI (E234:E257, salmon) has a kink at the highly conserved GlyE235 and GlyE236 and is prolonged as helix VIa (E224:E232) in the cytoplasm. The Na^+^ ions probably reach and leave the binding pocket by an entry at SerE35, AsnE36, GluE61, and SerE183 towards the MtrCDE cavity connected to bulk solvent (green arrow). Bonds between Na^+^ and their ligand are drawn as black lines and hydrogen bonds as red dashed lines. The Na^+^ binding pocket is located close to the CoM binding site next to Asn36 and the pore-forming helixes II and V. Disruption of the Na^+^ binding pocket may change the conformation of helix V of MtrE perhaps via the protonation state of GluE25. (*D*) Scheme of Na^+^ coordination. One Na^+^ (green) can be identified with high confidence (*SI Appendix*, Fig. S11), whereas the assignment of a second Na^+^ (light green) is rather speculative. The amino acids coordinating Na^+^ are highly conserved (*SI Appendix*, Fig. S7*E*).

Putative Na^+^ and CoM bind next to the cytoplasmic membrane boundary associated with a cavity formed by loops of helices II and V of MtrC and MtrD as well as those of helices II, III, and V of MtrE (Fig. 4*A*). The MtrCDE cavity has a diameter of 10 to 15 Å at the entrance and a depth of ca. 15 Å.

A cryo-EM map of MtrABCDEFG of *M. marburgensis* supplemented with 2 mM CoM prior to vitrification (Table 1 and *SI Appendix*, Fig. S10, EMD-18162, PDB-code: 8Q54) revealed the CoM binding site at the wall of the MtrCDE cavity (Fig. 4*A*). A continuous density of ambiguous profile was interpreted as two partially occupied CoM sites (Fig. 4*B*). In both sites, the sulfonate of CoM interacts with the conserved ArgE113 and AsnE38, while the thiol group is either attached to the cavity wall and hydrogen-bonded with AsnC188 or points into the bulk solvent. In contrast, MtrABCDEFG devoid of CoM contains two clearly separated solvent peaks at the positions of the sulfonate and the AsnC188-bound thiol group.

Na^+^ is most likely localized in a side pocket of the cavity ca. 10 Å away from CoM (Fig. 4*A*). Owing to the high resolution of the cryo-EM map (*SI Appendix*, Fig. S11), one metal ion could be reliably identified at the N-terminal ends of helices II (E61:E82) and VI (E234:E257) and the C-terminal ends of helices I (E3:E32) and V (E167:E182); the segments are characterized by special structural features described in Fig. 4*C*. We assigned the metal to Na^+^ on the basis of the presence of 80 mM NaCl in the enzyme solution prior to grid vitrification and the measured low K_d_ of 50 μM for Na^+^ (15) that implicates a high-affinity binding site. The Na^+^ ion is octahedrally coordinated by the mostly conserved SerE29, GluE61 and AspE187 as well as three water molecules (Fig. 4*D* and *SI Appendix*, Fig. S11). AspE187 has previously been predicted to be involved in Na^+^ transport (9). The Na^+^ binding pocket is sufficiently large to host a second Na^+^. The density between the highly conserved AspE26 and AspE30 is, however, too weak for a safe assignment of a metal ion as opposed to a solvent molecule (*SI Appendix*, Fig. S11). Further ligands of the uncertain second Na^+^ are GlyE186-O and H_2_O molecules (Fig. 4 *C* and *D*). Asp/Glu, Ser/Thr, and backbone carbonyl oxygens found as Na^+^ ligands are also reported for other Na^+^ translocating proteins (35–38). SerE35, AsnE36, and SerE183, residues of the cavity wall, are in direct contact with residues coordinating the Na^+^ ions (Fig. 4*C*). While the binding pocket is occluded in the metal-bound state, minor chain rearrangements in the Na^+^-free state would allow access for Na^+^ from the cavity.

### Models of the MtrA_s_–MtrH and MtrA_s_–MtrCDE Complexes.

The single-particle cryo-EM map only provides information about the MtrA_c_BCDEFG core complex of *M. marburgensis*, while structures of MtrH and MtrA_s_ are, so far, not experimentally accessible. Alphafold2 models (39) of MtrH from *M. marburgensis*, *M. wolfeii*, *Methanosarcina barkeri*, *Methanopyrus kandleri*, *Methanocaldococcus jannaschii,* and *Methanospirillum hungatei* consistently reveal a TIM barrel fold (*SI Appendix*, Fig. S12) as described for several other vitamin B_12_-dependent H_4_F/H_4_MPT-dependent methyltransferases (40–44). For all sequences tested, MtrH oligomerizes to a homodimer (*SI Appendix*, Fig. S12) as found for MtgA of *Desulfitobacterium hafniense,* the nearest relative of MtrH that has been structurally characterized (45). The Alphafold2 model of MtrA_s_ of *M. marburgensis* is essentially identical to the X-ray structure of the *M. jannaschii* protein and the *M. fervidus* MtrA homolog (21) (*SI Appendix*, Fig. S13). Therefore, B_12_ could be directly transferred to the MtrA_s_ of *M. marburgensis* from the superimposed MtrA homolog of *M. fervidus* hosting this prosthetic group. The distal ligand HisA84-NE2 is 2.9 Å apart from Co of the corrinoid ring, suggesting that the Alphafold2 MtrA structure is closer to a Co(III) (His-on) state. The expanded and bulk solvent-exposed segments downstream (A79-A83) and upstream (A96-A115) of helix A85:A94 are spatially neighbored to and firmly linked to HisA84 and thus conformationally susceptible to its repositioning upon transition between Co(I) and CH_3_-Co(III) (*SI Appendix*, Fig. S13). The MtrA_c_ helix, fixed as part of the stalk, and the mobile MtrA_s_ domain are connected by a conserved linker rich in glutamates and glycines (*SI Appendix*, Fig. S7*A*) that is disordered and thus like MtrA_s_ not visible in the cryo-EM map.

**Fig. 5.**
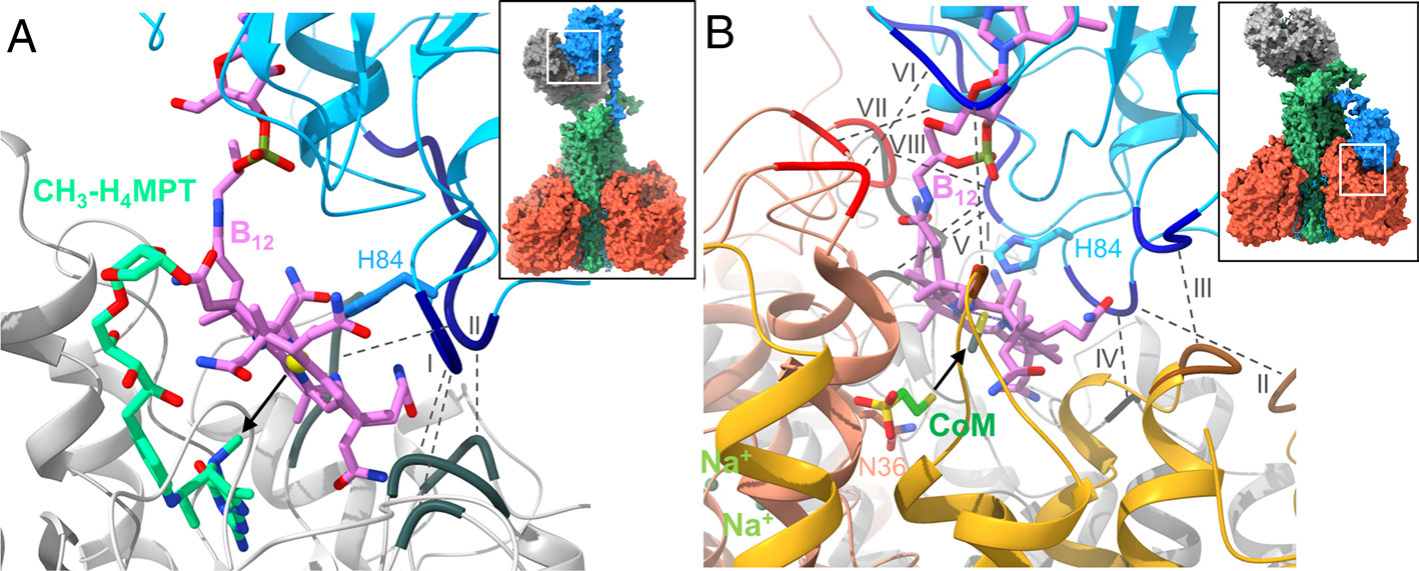
Alphafold2-based models of the MtrA_s_–MtrH and MtrA_s_–MtrCDE subcomplexes. (*A*) The active site geometry of the methyl transfer between methyl-H_4_MPT and cob(I)amide. The modeled corrinoid ring (carbon in pink) fits well into the opening of the MtrH TIM-barrel, the binding site of methyl-H_4_MPT (carbon in green). The black arrow indicates the direction of the attack of Co(I) onto the methyl group. Two MtrA_s_–MtrH interfaces are formed between the HisA84-associated segments (dark blue) and C-terminal loops of TIM barrel strands (black). Interactions occur between A82–A83 and H79 + H104–H105 (I) as well as A111–A115 and H132–H133 + H155–H158 (II). (*B*) The active site geometry of the methyl transfer between methyl-cob(IIII)amide (His-on) (carbon in pink) and CoM (carbon in green). The corrinoid ring of B_12_ bound to MtrA_s_ (blue) covers the cavity of MtrCDE (gold, light-gray, salmon). The direction of the nucleophilic attack of the CoM thiol sulfur (yellow) onto the methyl group (black) of CH_3_-cob(III)amide is marked by a black arrow. MtrA_s_ (dark blue)-MtrCDE (brown, black, red) interfaces are between segment A17 and C193 (I), A82 and C126–C127 (II), A33–A35 + A79 and C64 -C66 (III), perhaps A82–A83 and D192 (IV), A112–A114 and D35 + D122–D124 (V), A16–A17 and E119–E120 (VI), A56 and E47 (VII) as well as A112–A116 and E207–E208 (VIII). For orientation, the postulated overall structure of the two MtrABCDEFGH states (for details see *SI Appendix*, Fig. S15) are provided in the *Insets* (MtrCDE, red; MtrA_c_BFG stalk green; MtrA_s_, blue; MtrH, gray). The active sites are highlighted as white squares.

Subcomplexes of MtrA_s_–MtrH and MtrA_s_–MtrCDE have to be formed, at least transiently, to transfer the methyl group of methyl-H_4_MPT bound to MtrH to cob(I)amide of MtrA, and from there to CoM in the MtrCDE cavity. An Alphafold2 model of the MtrA_s_–MtrH_2_ subcomplex from various sequences approximately corresponds to the structural arrangement reported for other vitamin B_12_-binding protein—H_4_F/H_4_MPT-dependent methyltransferase complexes (Fig. 5*A* and *SI Appendix*, Fig. S14*A*) (41, 42, 44). The corrinoid ring, exposed in free MtrA_s_, projects into the TIM barrel of MtrH and interacts with several protruding loops of MtrH involving residues H103–H104, H109, H131–H133, H153–H157, and H199–H200 (*SI Appendix*, Fig. S7*H*). MtrA_s_ exclusively contacts MtrH via its HisA84-associated segments. The experimental data do not provide a conclusive indication, whether the Mtr(A_c_BCDEFG)_3_ core complex binds or does not bind to MtrH in the state when the latter does not form a complex with the mobile MtrA_s_ domain (Fig. 5*A*). Its presence as soluble protein in the cytoplasm (23) would argue for the former. On the other hand, Alphafold2 predictions on diverse MtrBH_2_ containing sequences [e.g., Mtr(A_c_BFG)_3_H_2_] reliably indicate an interface between MtrH and the N-terminal segment of MtrB that is disordered in the cryo-EM structure. A composite model made by aligning the cryo-EM model of (MtrA_c_BCDEFG)_3_ and AlphaFold2 models of MtrBH_2_ and MtrA_s_H_2_ is outlined in SI *Appendix*, Fig. S15*A*.

Sequence comparison and qualitative surface profile analyses conveyed a consistent hypothesis about the MtrA_s_–MtrCDE subcomplex in agreement with Alphafold2 predictions performed with sequences from various methanogenic archaea (Fig. 5*B* and *SI Appendix*, Fig. S14*B*). Accordingly, MtrA_s_ binds in front of the MtrCDE cavity next to the pseudo-threefold axis already described as the entry site for CoM and Na^+^ from the cytoplasmic side (Fig. 4). The modeled position of the B_12_ cofactor places the corrinoid ring in a way that it would completely cover the entrance of the cavity by interacting with segments E39–E43 and E207–E210 of MtrE, D28–D30 and D192–D194 of MtrD as well as C66–C69 and C191–C193 of MtrC localized at the cytoplasmic ends of helices II and V (Fig. 5*B*). Segment E44–E47 faces the nucleotide tail of B12. The MtrA_s_–MtrCDE interface depicted by AlphaFold2 and the proposed role of B_12_ in occluding the active site are supported by the high conservation of involved amino acids of MtrA_s_ and MtrCDE (*SI Appendix*, Fig. S7 *A*, *C*, *D*, and *E*) and the catalytic activity of exogenous B_12_ (15), which apparently binds to MtrCDE in the same manner. In addition, the appropriate length of the MtrA linker to reach the MtrCDE cavity from the stalk provides supporting evidence for the reliability of the model (Fig. 5*B* and *SI Appendix*, Fig. S15*B*).

### Substrate Binding and the Catalytic Process.

For exploring the structural basis of the methyl transfer between methyl-H_4_MPT and cob(I)amide, methyl-H_4_MPT was modeled into MtrH of the MtrA_s_–MtrH subcomplex by using the methyl-H_4_F binding of MtgA as template (45). Accordingly, the pterin head sits at the bottom of the TIM barrel of MtrH and the benzoyl, desoxyribose, and ribose moiety extends along the corrinoid ring of MtrA and helix H226-H233 to the protein surface (Fig. 5*A*). Highly conserved AspH195, AsnH128, AsnH229, and AspH102 (SI Appendix, Fig. S7*H*) are within hydrogen-bonding distance of the polar atoms of the modeled pterin molecule N2, N1, OD1, N5, and N8 thereby supporting the plausibility of the model. Co of the Co(III) (His-on)-like state and the methyl group of modeled methyl-H_4_MPT are rather far from each other (5 to 7 Å) in the MtrA_s_-MtrH subcomplexes (Fig. 5*A*). We assume speculatively a shorter distance in the Co(I) (His-off) state because HisA84 is repelled from Co(I) and thus sequestered together with the HisA84-associated segments contacting MtrH in the Co(III) (His-on)-like state. As a consequence, the corrinoid ring and residual MtrA_s_ can be placed closer toward MtrH without collision and the electron-rich Co(I) can attack the methyl group of methyl-H_4_MPT by a nucleophilic reaction. Concomitantly, HisA84 and the HisA84-associated segments are shifted towards Co(III) to form an octahedral methyl-Co(III) (His-on) state thereby interfering with MtrH. The MtrA_s_–MtrH subcomplex dissociates.

The Alphafold2-based model of the MtrA_s_–MtrCDE subcomplex offers information about the second methyl transfer reaction from methyl-cob(III)amide (His-on) to CoM. Binding of MtrA_s_ occludes the MtrCDE cavity with the corrinoid ring (Fig. 5*B*) and the position of the methyl group, projected to the cavity bottom, should be in the vicinity of the binding site of CoM (Fig. 5*B*). In fact, the distance between the methyl and thiol sulfur is ca. 5 Å when superimposing the MtrA_s_–MtrCDE Alphafold2 model and the experimental MtrABCDEFG-CoM complex (Fig. 5*B*).

**Fig. 6.**
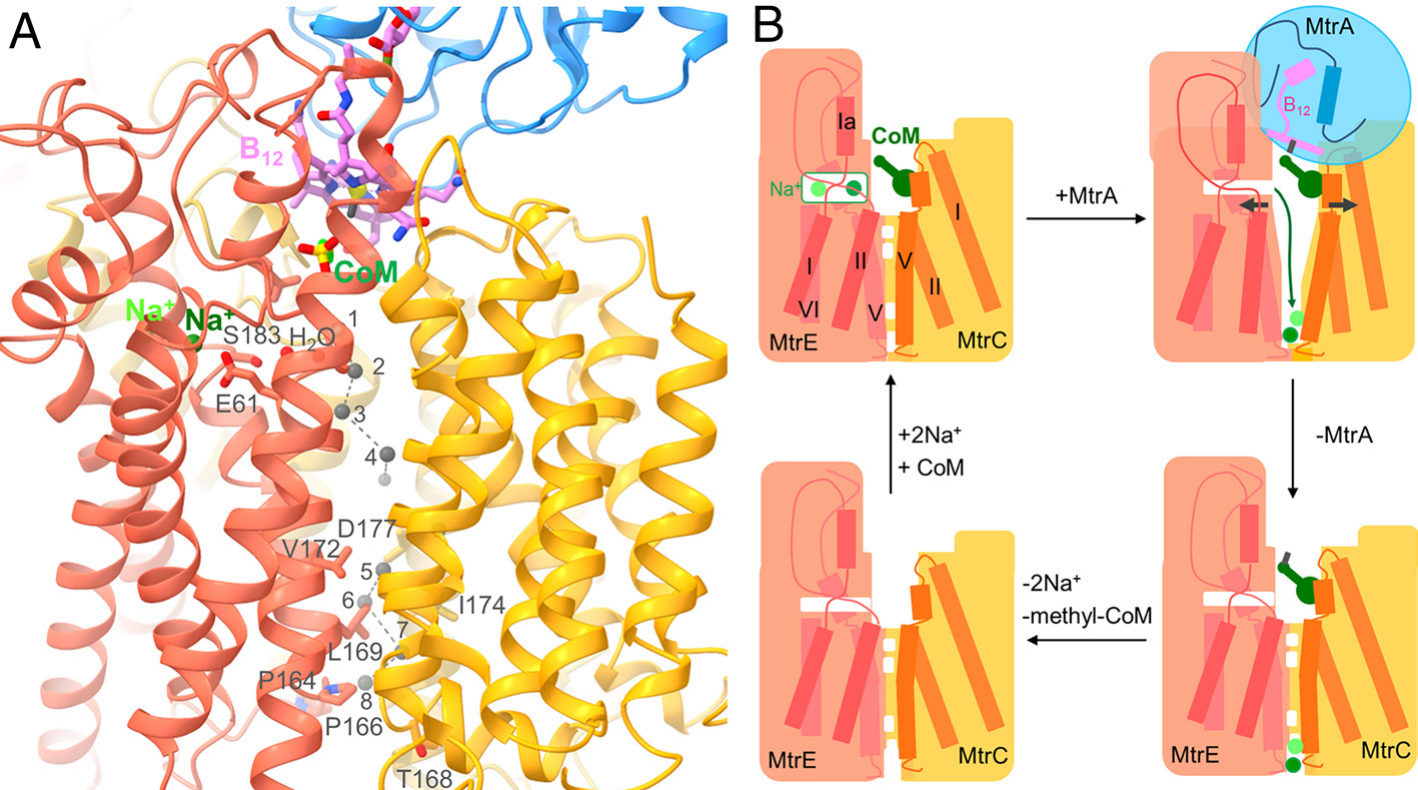
The methyl transfer coupled Na^+^ translocation. (*A*) The Na^+^ transmembrane pathway. The corrinoid ring (carbon in pink) exposed from MtrA_s_ in the isolated state occludes the MtrCDE (gold, yellow, tomato) cavity at the pseudo-threefold axis. CoM (carbon in green) binds to the wall and 1 or 2 Na^+^ (green) into a side pocket of the MtrCDE cavity. E61 and S183 mark their entry. Two experimentally found H_2_O molecules trace the path from the Na^+^ binding site to the postulated pore. Open sites in the pore across the membrane monolayer are filled by gray spheres as predicted by the Porewalker program (46). In the experimentally characterized Mtr(ABCDEFG)_3_ structure a barrier is found between sites 4 and 5. The narrow extracellular pocket sits around site 6. (*B*) Cartoon model of the proposed reaction cycle. The mechanism is based on the association of the corrinoid ring of methyl-cob(III)amide (His-on) and the dissociation of the cob(I)amide (His-off) carrying MtrA_s_ and the conformational changes induced thereby. The postulated scenario is compatible with the reversibility of the process.

On this basis we postulate a mechanism for the unique Na^+^ pump (Fig. 6): Na^+^ and CoM migrate to their binding sites via the open MtrCDE cavity (representing the experimental MtrABCDEFG-CoM complex structure). MtrA_s_ swings over and impinges with its exposed methyl-cob(III)amide (His-on) onto the rims of the MtrCDE cavity built up by loops up/downstream of the membrane-spanning helices II and V (Fig. 6). The intracellular helix Ia (E36:E44), placed next to the cobamide nucleotide tail, the neighbored helices I (E3:E32), II (E61:E82), and V (E167:E182) become rearranged and the Na^+^ binding pocket is thereby disrupted. Na^+^ is released into the MtrCDE cavity alongside SerE35, AsnE36, GluE61, SerE183, and AspE187 (from where Na^+^ originally entered its binding pocket) but cannot escape into bulk solvent in the presence of CoM and the MtrA bound corrinoid (Figs. 5*B* and 6*A*). Na^+^ can reach the pore gate along the pseudo-threefold axis at the bottom of the intracellular MtrCDE cavity (Fig. 6*A*), which might be opened due to outward forces on helices II and V imposed by MtrA_s_–MtrCDE interactions and the dissociation of Na^+^ (Fig. 4*C*). Under the guidance of the Porewalker program (46) executed with the experimental closed pore conformation, a constriction was localized at ca. two-thirds of the way (from the cytoplasmic side) across the membrane monolayer at ValE172, LeuE173, PheC176 and ValD177 (Fig. 6*A*) that might be removed after the outward helix movements. This would allow Na^+^ to reach a narrow hydrophobic site of the pore next to GlyC173, IleD174, and LeuE169 indicated by the green arrow in Fig. 6*B*. This site appears to be almost accessible from the extracellular space in the experimentally determined state but might be closed, e.g., by an inward movement of helices V of MtrC, MtrD, and MtrE after pore opening at the cytoplasmic side. Due to the spatial proximity, Na^+^ release and accompanying structural rearrangements might, in parallel, adjust an optimal active site geometry for CoM methylation, which would be consistent with the biochemical observation that addition of Na^+^ increases enzymatic activity (14). E.g., AsnE36 might move toward the CoM thiol and helix II (E61:E82) toward the CoM sulfonate (Fig. 5*B*) such that the loosely bound CoM thiol is rotated around the sulfonate anchor toward the corrinoid methyl. Thereafter, the CoM sulfur attacks the methyl group of methyl-cob(III)amide (His-on) and forms methyl-CoM and cob(I)amide (His-off) in a nucleophilic substitution reaction. Due to the backward movement of HisA84 transmitted to its associated segments, the interactions between MtrA_s_ and the MtrCDE globe are weakened, the strained MtrCDE helices relax, and the MtrCDE pore closes on the intracellular side and opens on the extracellular side (outward facing conformation) perhaps between ThrC168, AlaD169, and ProE166 (Fig. 6). Na^+^ exits the protein to the extracellular side. Finally, MtrA_s_ is detached from the MtrCDE globe, methyl-CoM leaves the open MtrCDE cavity and helices I, II, V, and VI of MtrE are arrested after binding of Na^+^ (Fig. 4*C*) thereby restoring the experimentally determined state.

The described cryo-EM and Alphafold2 model-based scenario outlines the first approximate mechanistic picture for the unique Na^+^ pumping methyltransferase reaction, although the detailed cascade of events, e.g., the driving force for charged Na^+^ (perhaps in a hydrated form) to enter the highly hydrophobic pore remains open. The structural data suggest an alternate access barrier mechanism (47) for Na^+^ translocation (Fig. 6*B*), by which the energy for cytoplasmic Na^+^ release and Na^+^ translocation is provided from an event outside the membrane region reminiscent to the situation reported for ABC transporters (48). The different ligation geometries of the Co(I) and CH_3_-Co(III) redox states appear to be the trigger (20, 49) to induce the dissociation/association of MtrA_s_, by which further processes are initiated (9, 22). B_12_ redox and ligation chemistry is, apparently, interposed for mechanistic reasons to physically accomplish the transformation of chemical into conformational energy. As a consequence, the overall redox neutrality of the two methyl transfers requires a complex multimodular protein with devices for executing the inverse redox reaction and for shuttling the corrinoid ring carrying MtrA_s_ between the two active sites. This shuttling function of MtrA_s_ over longer distances is in line with its disorder in the structurally determined state and the previously observed methylation and demethylation activities of exogeneous cob(I)amide and methyl-cob(III)amide, respectively (15). A related molecular juggling, required for Co(II) to Co(I) activation, Co(I) protection and methyl transfer, is reported for other B_12_-dependent methyltransferase systems (40).

## Materials and Methods

### Bacterial Strains, Media, and Growth Conditions.

*M. marburgensis* (DSM2133) and *M. wolfeii* (DSM 2970) were purchased from German Collection of Microorganisms and Cell Cultures (DSMZ). *M. marburgensis* was cultivated with a synthetic mineral medium as described previously (50) using a 10-L fermenter equipped with metal agitator parts. The fermenter was gassed with 80% H_2_/20% CO_2_/0.2% H_2_S at a flow rate of 4 L/min at 65 °C and an agitation of 1,000 rpm. We harvested the cells at late logarithmic phase and stored them at −75 °C. *M. wolfeii* was cultivated using a 1.5-L fermenter equipped with metal agitator parts. The fermenter contains the synthetic medium described previously (51) and was gassed with 80% H_2_/20% CO_2_/0.01% H_2_S at the flow rate of 0.8 L/min with an agitation of 1,000 rpm. The cells were harvested at late logarithmic phase and stored at −75 °C.

### Mechanical Cell Disruption and Membrane Solubilization of *M. marburgensis* and *M. wolfeii*.

Cells were suspended in lysis buffer [50 mM Mops/NaOH pH 7.0, 10 mM MgCl_2_ and 2 mM dithiothreitol (DTT)] in a 1:2 volume ratio, i.e., 1 part (30 mL) wet cell to 2 part (60 mL) buffer and disrupted by a French press (1,100 psi) three times on ice in a brown flask. The lysate was centrifuged at 15,000 rpm (26,892 g) for 30 min and the obtained supernatant at 45,000 rpm (235,000 g) for 90 min both at 4 °C. The membrane fraction was washed twice with lysis buffer and stored at −80 °C after flash-freezing in liquid nitrogen. Membrane pellet was solubilized in extraction buffer [50 mM HEPES/NaOH pH 8, 10 mM MgCl_2_, 1.5% w/v dodecyl-β-D-maltoside (DDM), 2 mM DTT], incubated for ca. 12 h at 4 °C under slight agitation. Finally, the resuspension was ultracentrifuged at 55,000 r.p.m. (340,000 g) for 2 h at 4 °C.

### Biological Cell Disruption and Membrane Solubilization of *M. marburgensis*.

For biological disruption, 12 g cells of *M. marburgensis* were dispersed in 24 mL suspension buffer (50 mM Mops/NaOH pH 7.0, 7.3 mM K_2_HPO4, 1 mM DTT, 5% glycerol and 2.5% DDM), heated at 60 °C for 10 min and supplemented with reaction buffer (50 mM Bis-Tris propane pH 7.0, 10 mM DTT and 10 mM MgCl_2_) in a 2:1 ratio. Finally, 200 μL recombinant pseudomurein endopeptidase (2.3 mg/mL) was added. The cell suspension was incubated at 25 °C overnight and then centrifuged at 10,000 rpm for 15 min. For complete separation of the membrane fraction from cell debris, the supernatant was again centrifuged at 50,000 rpm for 1.5 h. The membrane fraction was solubilized in the extraction buffer for ca. 12 h at 4 °C with a slight agitation and finally ultracentrifuged at 100,000 g (55,000 rpm) for 2 h at 4 °C.

### Purification of *M. marburgensis* and *M. wolfeii* MtrABCDEFGH.

First, 9.5 mL of 10 to 50% sucrose gradient solution (50 mM Hepes pH 8.0, 2 mM DTT, 10 mM MgCl_2_ and 1.5% DDM with 0 and 50% sucrose) was made in SW40 tubes, and 1.8 mL of the solubilized membrane fraction (approx. 100 μg/mL protein) was added to each tube. After overnight centrifugation at 35,000 rpm, the gradient solution was fractionated from top to bottom in ca. 1 mL fractions using glass pipettes. The fractions containing dark-pink MtrABCDEFGH complex eluted at a sucrose gradient of 30 to 40%.

After pooling and filtering the sample through a 0.45-μm filter (Sartorius), the enriched MtrABCDEFGH fractions were applied to a 10 mL DEAE Sepharose FF column preequilibrated with IEC-1 buffer (50 mM MOPS/NaOH pH 7, 10 mM MgCl_2_, 2 mM DTT, 100 mM NaCl, 0.05% w/v DDM) and eluted with a gradient from 0 to 1 M NaCl in IEC-1 buffer at approximately 340 mM NaCl. After pooling, diluting by a factor of 3 with IEC-1 buffer, and washing a 5 mL Q sepharose HP column with 3 column volume (CV) buffer IEC-1, the MtrABCDEFGH fraction was loaded, washed with 20 CV of IEC-2 buffer [50 mM MOPS/NaOH pH 7, 10 mM MgCl_2_, 2 mM DTT, 100 mM NaCl, 3 CMC lauryl maltose neopentyl glycol (LMNG)] and eluted with a gradient of 0 to 1 mM NaCl in IEC-2 buffer at 1 mL/min within 15 CV. The pink MtrABCDEFGH fraction was pooled and concentrated with a 50 kDa-cutoff centricon. Then, 500 μL sample was injected into a 0.5-mL loop of a Superose™ 6 Increase 10/300 GL column pre-equilibrated with 50 mM MOPS/NaOH pH 7, 10 mM MgCl_2_, 2 mM DTT, 80 mM NaCl, 3 CMC LMNG. The flow rate for gelfiltration was 0.3 mL/min. Related purification protocols were elaborated in the past (12, 22, 23).

### Protein Analysis.

Protein concentration was determined by Bradford (52). The integrity and purity of the protein complex were explored by SDS-PAGE analysis and mass-spectrometry. For mass-spectrometry, the gel bands were cut out, buffered, and trypsin (SERVA)-digested overnight. The peptide solution was acidified and subjected to liquid chromatography-mass spectrometry. This liquid chromatography - mass spectrometric analysis was performed using an Orbitrap Exploris 480 mass spectrometer connected to an Ultimate 3000 RSLC nano with a nanospray ion source (all Thermo Scientific). The peptide separation was carried out on a reversed-phase HPLC column (C18) within 30 min by using a separation gradient of 0.15% formic acid/acetonitrile. The data acquisition mode was set to include a high-resolution mass spectrometer (MS) scan, followed by MS/MS scans of the most intense ions. The MS data were interpreted with Proteome Discoverer 1.4.

### Cryo-EM Specimen Preparation, Data Collection, and Image Processing.

Cryo-EM grids of MtrABCDEFGH were prepared using Quantifoil CF-1.2/1.3-3C, Cu/C-50 grids (300 mesh, 1.2-µm hole size). After glow-discharging for 90 s at 15 mA in an easiGlow system, the grids were loaded with 3 µL of the sample (concentration 2 mg/mL), blotted using Whatman 595 blotting paper (Sigma-Aldrich) for 3 to 4 s, and plunge-frozen in liquid ethane using a FEI Vitrobot IV maintained at 4 °C and 100% humidity.

Movies were recorded on a Titan Krios G3i microscope operated at 300 kV (Thermo Scientific), which was equipped with a K3 direct electron detector and a Gatan BioQuantum imaging filter using a 30 eV slit width. Data collection was executed at a nominal magnification of 105,000× in electron counting mode using aberration-free image-shift correction in EPU (Thermo Scientific). Applied parameters are listed in Table 1. The full dataset was processed with RELION-3.1 (25, 53). Beam-induced motion was corrected with MOTIONCOR2 (54) and dose-weighted images were generated from movies. Initial contrast transfer function parameters for each movie were estimated using Gctf algorithms (55). Particles were picked with crYOLO (56) using the neural network-trained general model approach. 2D classification, unsupervised initial model building, 3D classification, 3D refinement along with post-processing steps like contrast transfer function refinement, Bayesian polishing, and final map reconstructions were performed with RELION-3.1 applying C3 symmetry. Density modification was performed in PHENIX (27). Maps were visualized with Chimera (57) and COOT (58). Models were automatically built with Arp/Warp (59) or manually within COOT. Twelve tetraether glycolipids, 2 Na^+^, 1 Mg^2+^, and a larger number of water molecules were integrated into the model, which was subjected to the real-space refinement tool of PHENIX. The occupancy of lipid atoms that were unclear in the density was set to 0. In a second data set (Table 1), MtrABCDEFGH was supplemented with 2 mM CoM and treated as described above. Density for CoM could be identified at a plausible site. Model quality was assessed with MolProbity (60) in PHENIX.

## Supplementary Material

Appendix 01 (PDF)

## Data Availability

The cryo-EM density maps of the Mtr and the Mtr-coenzyme M complexes have been deposited in the Electron Microscopy Data Bank (EMDB) under accession code EMD-18135 (https://www.ebi.ac.uk/pdbe/entry/emdb/EMD-18135) (61) and EMD-18162 (62). The coordinates have been deposited in the RCSB Protein Data Bank (PDB) under accession code 8Q3V (https://doi.org/10.2210/pdb8Q3V/pdb) (63) and 8Q54 (64). All other data are included in the manuscript and/or *SI Appendix*.
