# Supplementary material for: Structural and mechanistic basis of the central energy-converting methyltransferase complex of methanogenesis
Source: Proc Natl Acad Sci U S A. 2024 Mar 26;121(14):e2315568121. doi: 10.1073/pnas.2315568121 (PMC10998594; doi:10.1073/pnas.2315568121)
Supplement: Supplementary file 1 — Appendix 01 (PDF) [file pnas.2315568121.sapp.pdf]

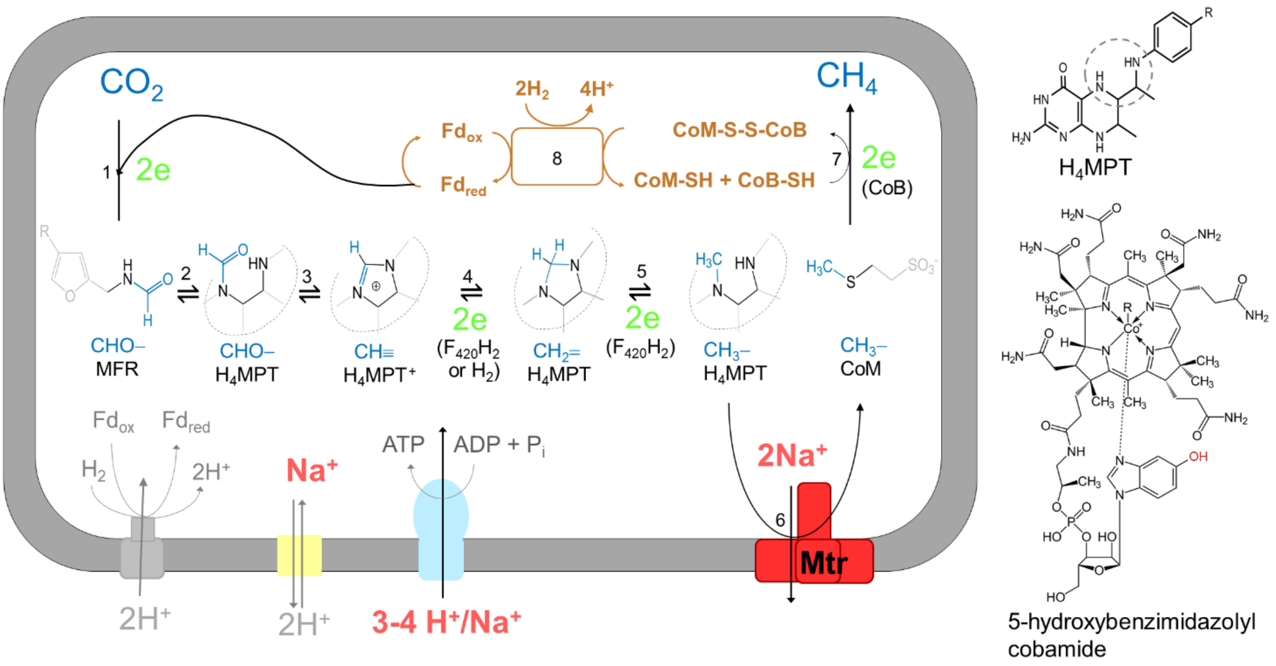

Fig. S1. Methanogenesis. Methanogenic archaea use fermentation products as carbon dioxide, methanol or acetate as substrates for performing the final process of anaerobic biomass degradation (1, 2). In the hydrogenotrophic variant of methanogenesis (3, 4) (shown here) carbon dioxide (CO_2) is stepwise reduced to methane (CH_4). Reactions 2 - 5 are equilibrium reactions. Reaction 8, the exergonic CoM-S-S-CoB reduction to CoM and coenzyme B drives reaction 1, the endergonic CO_2 reduction, via a flavin-based electron bifurcation process (5-7). Reaction 6, the exergonic methyl transfer from methyl-H_4MPT to CoM coupled with Na^+ translocation is catalyzed by MtrABCDEFGH (Mtr, red). The membrane protein carrying the vitamin B_{12} derivative 5-hydroxybenzimidazolyl cobamide catalyzes the only electrogenic process in the pathway, which is used for ATP synthesis by ATPase (blue). A membrane-spanning [Ni,Fe] hydrogenase (gray) supplies the cycle with additional reduced ferredoxin to maintain a continuous operation when intermediates for the anabolism are withdrawn (3). In the methylotrophic variant (8) methanol, methyl amine and methyl thiol enter the pathway at the methyl-CoM state. From four methyl-CoM three are reduced to methane and one is oxidized backwards to CO_2. Acetate and methoxylated aromatic compounds enter their degrading pathways (8, 9) at methyl-H_4MPT. The methyltransferase reaction is not compulsory for the energy conservation of all methanogenic archaea. Methanomasiliiicoccales e.g. *M. stadtmanei* growing on methanol, are devoid of Mtr and use an alternative pathway variant (6, 10). Methane, the end product of the anaerobic food web, is either anaerobically or aerobically oxidized to CO_2, escapes into the atmosphere as greenhouse gas, or accumulates as methane hydrate in the deep sea.

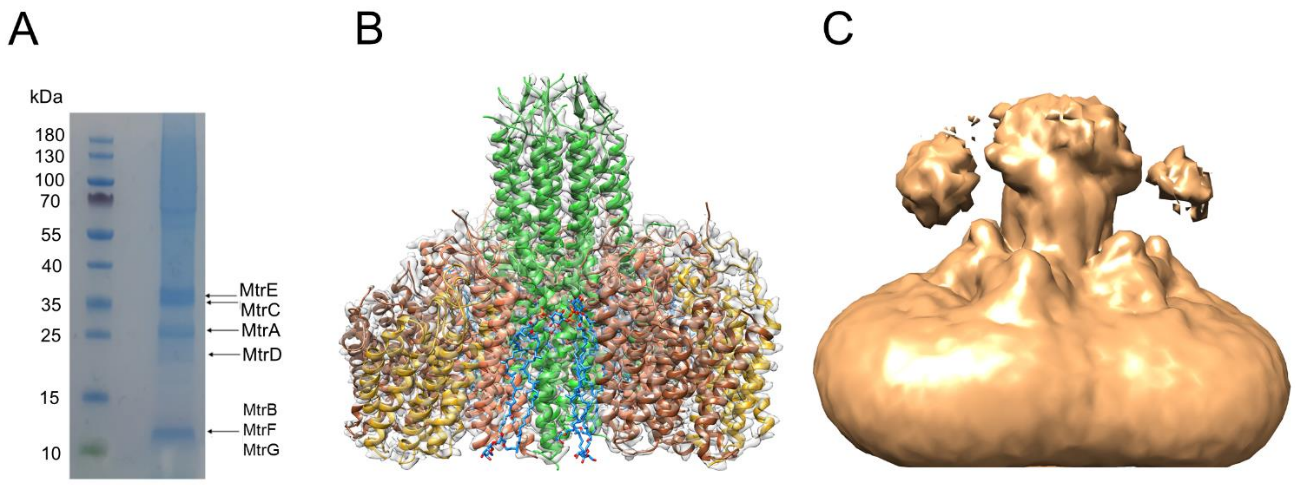

Fig. S2. The MtrABCDEFG subcomplex of *M. marburgensis* devoid of MtrH after chemical removal. (A) SDS-PAGE of the purified enzyme. The individual bands were tentatively assigned according to the molecular masses of the subunits (MtrA 25.6 kDa, MtrB 10.7, MtrC 27.1, MtrD 22.8, MtrE 31.2, MtrF 7.3, MtrG 9.5, and MtrH 33.5). For preparation *M. marburgensis* cells were disrupted with a French press. MtrH was removed from the MtrABCDEFG(H) complex by chemical modification using dimethyl maleic anhydride (DMMA) and solubilized with 2.5% n-dodecyl-β-D-maltoside (DDM) as reported previously (11). The MtrABCDEFG complex was purified by DEAE- and Q-sepharose ion exchange chromatography and Superose-6 size exclusion chromatography. For grid preparation only protein of the peak fraction was applied. Detergent exchange was performed during the Q-sepharose chromatography step by eluting with 50 mM MOPS/NaOH pH 7, 10 mM $MgCl_2$, 2 mM DTT, 100-1000 mM NaCl and 3 CMC GDN (glyco-diosgenin). (B) Cryo-EM map (gray surface) and superimposed model at 2.37 Å resolution. The structures based on preparations with and without MtrH are virtually identical. The rms deviations are 0.3 Å. Despite differences in purification and detergent solubilization the found tetraether lipids and their positions remain nearly unchanged thereby documenting the high protein-lipid affinity. This also holds true for the different occupation of the two putative Na^+. (C) The cryo-EM map after 3D classification. Using Relion (12) a map was calculated at 6.9 Å resolution based on 103947 particles without applying the threefold symmetry. Three density pieces (one is behind the stalk) are visible above the noise level and might image the three $MtrA_s$ domains.

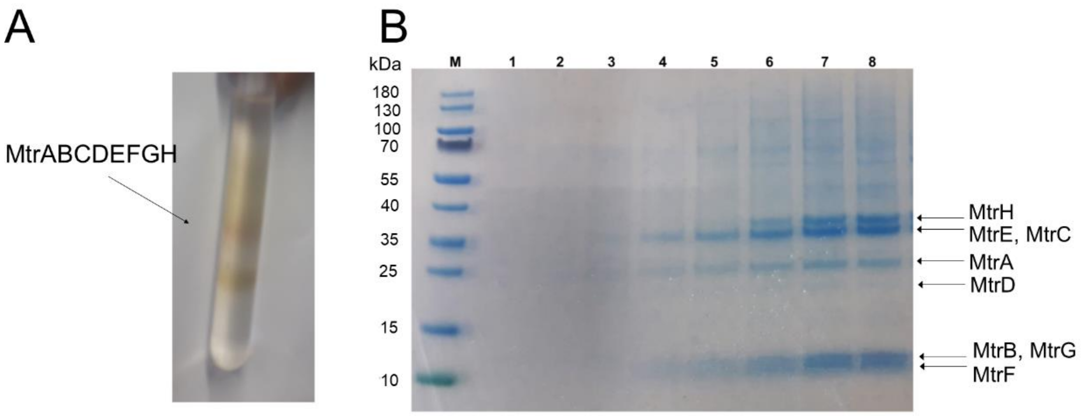

C

	unique peptides	peptides	PSMs
MtrA	14	17	473
MtrB	2	4	113
MtrC	2	3	45
MtrD	2	2	71
MtrE	3	3	132
MtrF	6	8	140
MtrG	7	9	140
MtrH	20	23	345

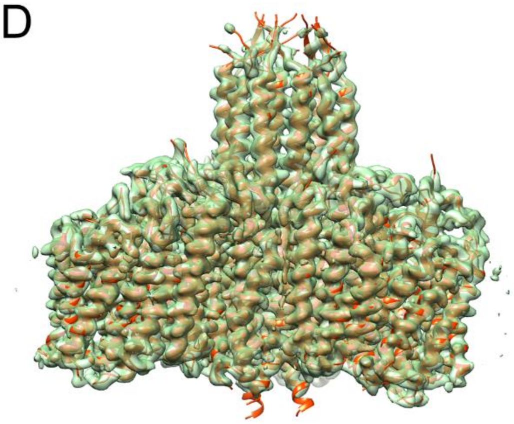

Fig. S3. MtrABCDEFGH of *M. wolfeii*. (A) The enzyme complex after sucrose gradient centrifugation. The pink color of the protein indicates the presence of MtrA$_s$. (B) SDS-PAGE analysis after the final gel filtration step using a Superose TM6 Increase 10/300 GL column. (C) Liquid chromatography mass-spectrometric (LC-MS) analysis. For preparation, the gel bands were cut out, buffered and trypsin (SERVA) digested overnight. After acidification, the obtained peptides were applied to a C18 solid phase extraction column, eluted with 50% acetonitril/0.1% trifluoroacetic acid, dried and reconstituted in 0.1% trifluoroacetic acid. PSMs describes the total number of the identified peptide spectra that match the MtrABCDEFGH sequence. LC-MS data confirmed the presence of all eight subunits. The most frequently found subunits are MtrH and MtrA indicating that vitrification was started from a sample containing populations of the intact MtrABCDEFGH complex. (D) Cryo-EM map at 3.3 Å resolution. The cryo-EM density map (green surface) excellently fit with the superimposed model of the MtrA$_c$BCDEFG complex. The soluble MtrA$_s$ domain and MtrH were not visible. The structures of MtrA$_c$BCDEFG complex of *M. marburgensis* and *M. wolfeii* were virtually identical; the rms deviation is 0.43 Å by a sequence identity of 95%.

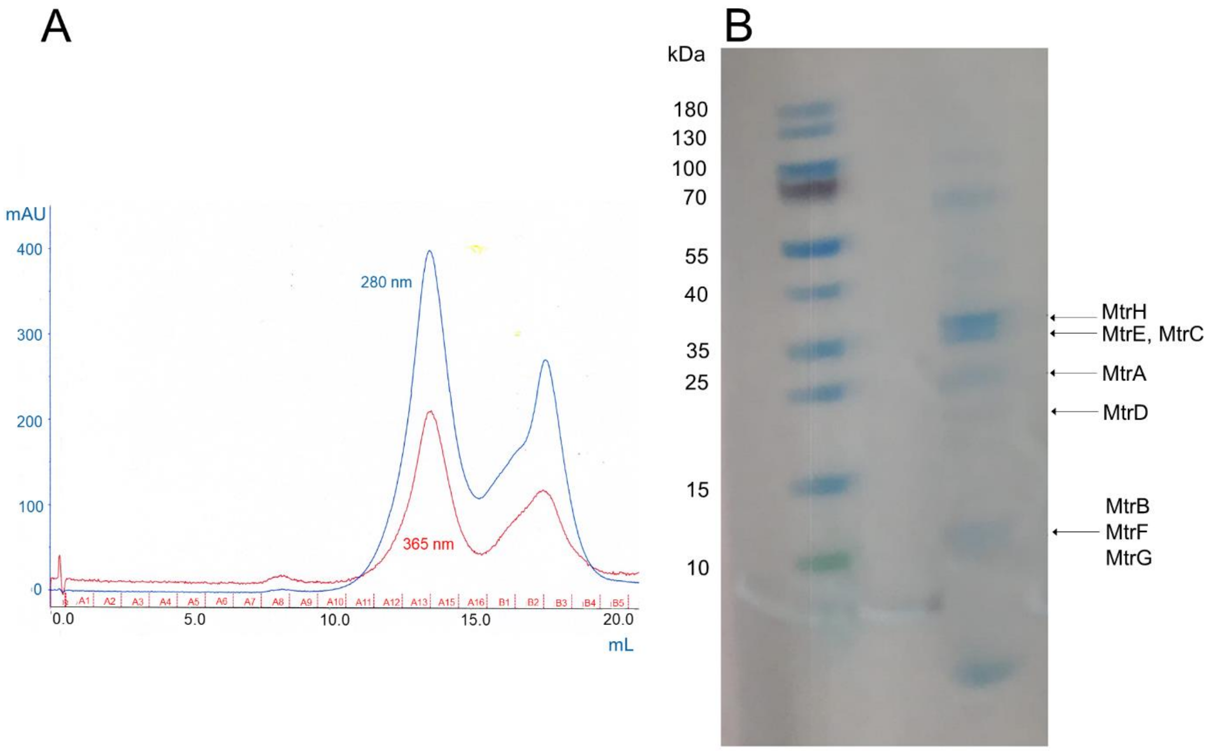

Fig. S4. Biochemical analysis of the MtrABCDEFGH complex of *M. marburgensis*. (A) Elution profile of the final size exclusion chromatography. The protein complex was eluted after 13.5 ml retention volume from the Superose TM6 Increase 10/300 GL column. Only protein of the peak fraction was applied for grid preparation. The experiment was done 5 times with a yield of ca. 1 mg MtrABCDDEFGH from 30 g cells. Due to the limited amount of sample the B_{12} content and the Co oxidation state were not explicitly measured. We estimated the B_{12} content as rather high due to SDS-PAGE and mass-spectrometric data and the cob(III)amide portion as, unexpectedly, high due to the pink color and the high peak height at 365 nm relative to that at 280 nm. (B) SDS-PAGE after the final Superose TM6 gel filtration. Bands are highly similar in the *M. marburgensis* and *M. wolfeii* enzymes.

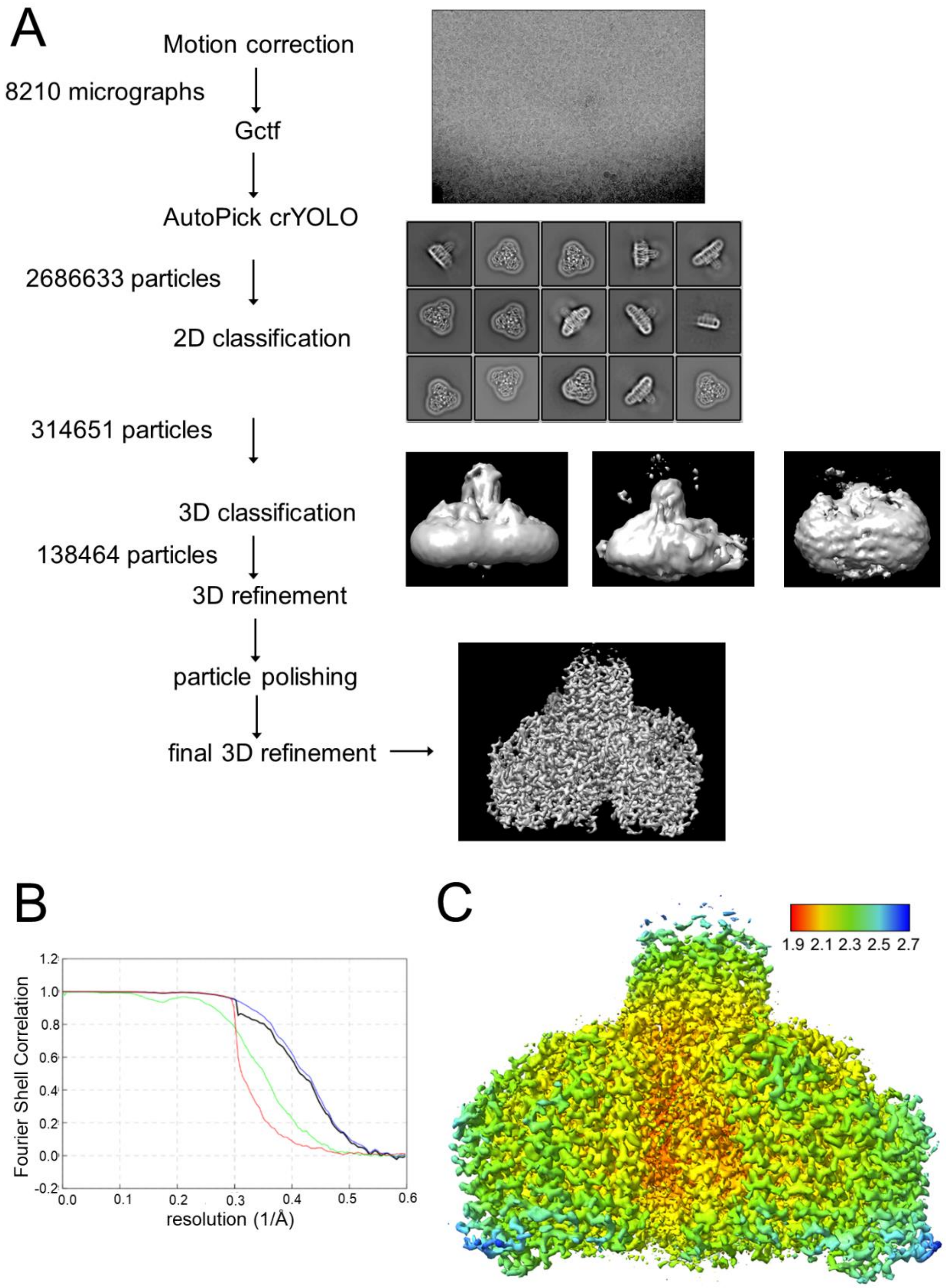

Fig. S5. Cryo-EM Mtr(ABCDEFG)$_3$ structure determination using Relion. (A) Workflow of data processing including a micrograph image, as well as 2D/3D classification and 3D refinement maps**.** Data collection was done on a Titan Krios EM at 300 kV equipped with a Gatan K3 detector using a pixel size of 0.837 Å at 105000x magnification. (B) Gold-standard FSC plot (green curve: unmasked; blue: masked; red, phase randomized masked; black, FSC corrected for overfitting). Resolution estimated at FSC=0.143. (C) Map of the protein complex viewed parallel to the membrane plane colored by local resolution.

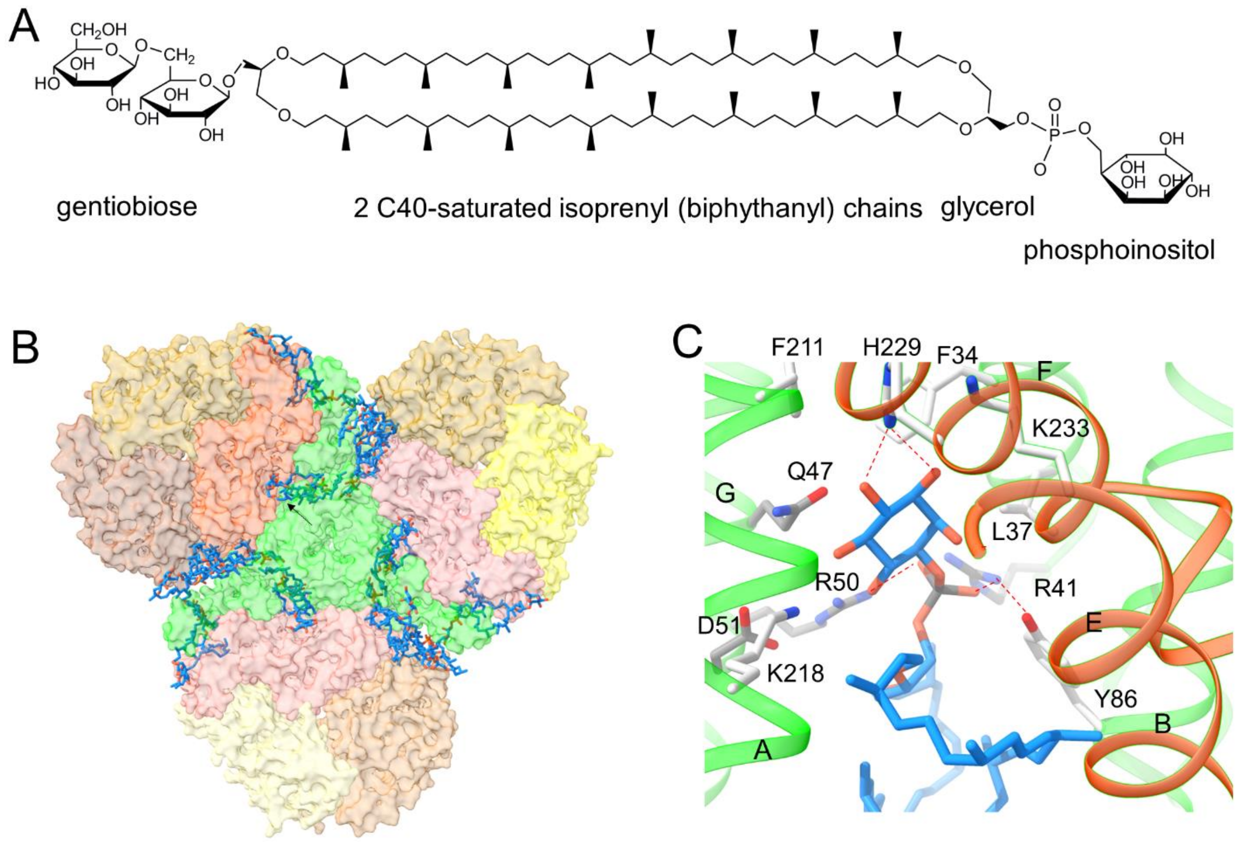

Fig. S6. Tetraether glycolipids. (A) Chemical structure. The cryo-EM structure of MtrABCDEFG revealed several C40 biphytanyl chain-derived lipids. We assigned them as gentiobiosyl - glycerol diacyl glycerol tetraether (GDPT) - phosphoinositol (13, 14), the most abundant tetraether glycolipid in *M. marburgensis* according to mass-spectrometric data (15). Additional methylations of the saturated isoprene chain and modifications of the polar heads (16, 17) were not obvious in the density and thus not taken into accounts. Some of the lipids are well ordered (Fig. 1D), others adopt multiple conformations and their densities overlap with that of partner lipids. The terminal glycoside at the gentiobiosyl head is disordered in all lipids and was therefore not modelled. (B) Lipid binding sites in the Mtr(ABCDEFG)$_3$ complex. Four of the 12 tetraether glycolipids (carbon in blue) are neighbored to each other thereby filling out the space between two MtrCDE globes (brown, yellow, red) and between the MtrBF and MtrAG helices of the stalk (green). The most interior tetraether glycolipid (see back arrow) is the one best defined (Fig. 1D). Tetraether lipids crossing the membrane in a rather natural manner are, to our knowledge, not characterized in the structures of membrane protein complexes but in hydrophobic clefts between tail and capsid of a spindle-shaped archaeal virus (18) and as substrate in tetraether lipid synthase (19). (C) Binding of a phosphoinositol head. The cytoplasmic surface of the membrane is characterized by positively charged residues (20) that form hydrogen bonds with the phosphate group of the lipids. The interacting ArgG50 is strictly conserved, ArgF41 is variable. The structural data indicate that lipids play a crucial role to connect different subunits and thus to increase the stability of the multi-modular complex.

A

```
WP_011977196.1    M.mari     MANKKSPAATWPVANGEYVLGNPESCVGVITLGSHGLDQAAVDAG---AALSGPCHTENL    57
WP_064496640.1    M.jann     MANKREPAPGWPIVSGEYVVGNPESCVGVVTLGSHGLEQACIDAG---AAIAGPCHTENL    57
WP_013867105.1    M.okin     MADKKEPAAGWPIVSGEYVVGNPESCVGVVTLGSHGLDDAAISAG---AAISGPCHTENL    57
CAA74771.1        M.kand     MPEKAEPAEGWPVVEGDYVVGDPEAPVHVVTLGSHIEEDILKAAGEDKVAIAGPCKTENI    60
ADL59122.1        M.marb     MVEKKSPAEGWPVVNGDYIVGDPESPVAATTLASHIEDIPV-EAG---AAIAGPCKTENL    56
NLM03298.1        M.wolf     MVEKKSPAEGWPVVNGDYIVGDPESPVAATTLASHIEDVPV-EAG---AAIAGPCKTENL    56
WP_011020330.1    M.acet     MVDKKEPASGWPILKGEYEVGDVKGCVAVITCASHLPGKPVLDAG---AAITGSCKTENL    57
WP_011306268.1    M.bark     MADKKEPAPGWPILKGEYEVGDVKNCVLVITCGSHLPGQPILDAG---AAVTGSCKTENL    57
WP_011033489.1    M.maze     MADKREPAPGWPILKGEYEVGDVKNSVLVITCGSHLPGKPILDAG---AACTGSCKTENL    57
WP_012618918.1    M.palu     MADKKSPASGWPIVQGDFHSGDANSPVAVITMGSHLDEQGICDAG---AAICGSCKTENL    57
QXO93993.1        M.hung     MANKKSPASGWPIVQGDFHTGDPNSPVAVVTMGSHLDEGAICSAG---AAIAGSCKTENL    57
                             * :* .**  **: .*::  *: :  * . * .**        **   .*  * *:***:

WP_011977196.1               GIEKVVANYISNPNIRFMIIAGSEVQGHITGQCIKALYENGIGDD-GGIIGAKGAIPFME    116
WP_064496640.1               GIEKVVANYISNPNIRFMILCGSEVQGHITGQCFKALWENGIGDD-GGIIGAKGAIPFLE    116
WP_013867105.1               GIEKVVANYISNPNIRFMVLCGSEVQGHITGQCFKALHENGIGDD-GGIIGAKGAIPFLE    116
CAA74771.1                   GIEKVIANVIANPNIRFGVLCGAEVTGHLTGQCFKAMYENGVDPDSGEIIGAEGAIPYLE    120
ADL59122.1                   GIEKMIANLISNPNIRFLILCGSEVQGHITGQSIEALHQNGVDPDKRNIIGATGAIPYIE    116
NLM03298.1                   GIEKMIANLISNPNIRFLILCGSEVQGHITGQSIEALHQNGVDPDKRNIIGATGAIPYIE    116
WP_011020330.1               GIEKVVAHVISNPNIRYLVVTGAEVKGHVTGQAMLSIHANGVKE--HRIVGAVGAIPYVE    115
WP_011306268.1               GIEKVVAHIISNPNIRYLLVTGSEVKGHITGQSVMSLHANGVKD--NRISGALGAIPYVE    115
WP_011033489.1               GIEKVVAHIISNPNIRYLLVTGSEVKGHITGQSMMSLHANGVKE--NRIAGALGAIPYVE    115
WP_012618918.1               GLEKVVANVISNPNIRFVLLCGTEVKGHLSGQSLRQLHSSGVDK--GKIVGSEGAIPFIE    115
QXO93993.1                   GLEKVIANVISNPNIRFVITCGTEVKGHLSGQSLIALHQNGVDG--GKIVGSKGAIPFIE    115
                             *:**::*: *:*****: :  *:** **::**..  :  .*:       * *: ****::*

WP_011977196.1               NIGKEPVERLQRQIIDCIDLIDVEDTAKIAAAIKNCTSQDPDAIDEEPMVVDLEGGEAVA    176
WP_064496640.1               NVNKEAVERFRRQIVEVVDLIDCEDIGKITQAIKECLSKDPGAIDEDPFIIELEGGKGGG    176
WP_013867105.1               NAGKDAIDRFRRQIVEIVDLIDCEDTGKISSAVKECISKDPGAIDEDPFIIELEGGAGGE    176
CAA74771.1                   NIPEEAVERYRDQIVELVDLIDVEDVDEIVKAIEECVEKDPGAYEEGPMTISLEEEEE-E    179
ADL59122.1                   NIPDEGIERFQKQL-EIVNLIDVEDADAIKAKVKECIEKDPGAFEEEAMIIKVEEGGE-E    174
NLM03298.1                   NIPDEGIERFQKQL-EIVNLIDVEDADAIKAKVKECIEKDPGAFEEEAMIIKVEEGGE-E    174
WP_011020330.1               NLNDDAVARFQQQI-ETVNLLDTEDMGAITAKVRELVAKDPGAFDAEPMVVEISEEGE-E    173
WP_011306268.1               NLNEDAIARFQEQV-DVVNLLDTEDMGAITSKVKELASKDPGAFDAEPMIVEISEEGE-E    173
WP_011033489.1               NLNAAAVARFQEQV-QVVNLLDTEDMGAITSKVRELASKDPGAFDADPLVVEISEEGE-E    173
WP_012618918.1               NLDDAAIKRFQEQI-EIVNIMESEDLGTIKAKINELKGKDPGAFAGDAMVVEVKEAGGAA    174
QXO93993.1                   NLSADNMKRFQEQV-EIVDIMESEDLGAISAKIKELTARDPGAFDAEAMVVEIKEEGGGA    174
                             *     : * : *: : ::::: **   *   :.:   :**.*      : :.:.

WP_011977196.1               NTESTSMKPTSPEMALLEARMKIVSEKMNEAAMIAKFNSGYYNGKIQGIAIGLFLSILVF    236
WP_064496640.1               EEEEGVIKPITPEMAIIESRMRLIGNEMCYNGLLAKWQAGYYNGKIQGIATGLFLMLLIM    236
WP_013867105.1               EEEGTAIKPITPEMAIVESRLRIISEAINYNALLAKWQSGYYNGKIQGISIGLFMSILVL    236
CAA74771.1                   ELAEVAGMPVSAETVTVEYRINDVRVGVKSIGAMQRYMAGYLSGRTMGLLIGIISGMIFL    239
ADL59122.1                   -EEGEEVKPVAPETALIEARMRNIQTQVKMIGSTNRMFAGMYSGKVQGIMIGLAFTLTLG    233
NLM03298.1                   EEEGEEVKPVAPETALIEARMRNIQTQVKMIGSTNRMFAGMYSGKVQGIMIGLAFTLTLG    234
WP_011020330.1               EEEGGVVRPVSGEIAVIRSRLKAIEARMLDIGNLNKFHSGVHAGKIEGAMIGLTVTISLL    233
WP_011306268.1               EEEGGAVRPVSGEIAVIRSRLKAIEARMMDIGNLNKFHSGVHAGKIEGAMIGLTVTISLL    233
WP_011033489.1               EEEGGVVRPVSGEIAVLRSRLKAIEARMMDIGNLNKFHSGVHAGKVEGAMIGLTITISLL    233
WP_012618918.1               EESTGEVQPLSGEVVLVHARMKVIEKMITDIGYWDRYAAGVYSGKVEGLMIGLIVSFALI    234
QXO93993.1                   AEEGGEVRPMSAEVALIHARMKTVQRMITDIGFYDKYAAGVYGGKVEGLMIGLIVSFVIV    234
                                     * : * . :. *:. :   :   .   :  :*   *:  *   *:   : .

WP_011977196.1               SLL-------          239
WP_064496640.1               GILMF-----          241
WP_013867105.1               GILMF-----          241
CAA74771.1                   FLPMVVLGGV          249
ADL59122.1                   ILLLV-----          238
NLM03298.1                   ILLLV-----          239
WP_011020330.1               GLLLLGR---          240
WP_011306268.1               GLLLLGR---          240
WP_011033489.1               GLLLLGR---          240
WP_012618918.1               GFLLMG----          240
QXO93993.1                   GLLLVGHGVS          244
                               :
```

B

```
WP_018153518.1  M.ther     ---MEIIKVCPELNVVMDVDSGLIAEM-RKDVLVVDLNPIEEEINKLEKLAKAFENSLDP    56
AVB76654.1      M.mari     ---MDIVKVCPELHIVMDVDSGLIAEM-RKDILVVDLHPVEDEINKLAQYAKALENSLDP    56
sp|Q58260.1|    M.jann     --MATYVFIDQNIPLVYTVETGVITKG-FGDLLFVDVSPIEEQIKKLETLVDAYEHSLDP    57
WP_013867106.1  M.okin     ---MAILYIDKEIPLVYNIENGKITKG-LGDVIFVDTDPINQQIDRLEEYVSSYEQSLDP    56
WP_048145368.1  M.palu     ---MMYVQVLPEYGLVLDPMVGIVTTA------GESFAPVLEQIAILESASDDLVNMLSG    51
QXO93994.1      M.hung     ---MTWIPVLPDFGLGCDVFAGFVTQQ------GESLAPIVEQVNKLEKVTDDIVGMLSG    51
CAA74770.1      M.kand     ---MAIVLIDPESQIAMDAVTGAVAEW-SEDVVTLDVMPLYEKVEELEQYVNDMMRAMDP    56
ADL59123.1      M.marb     MEMLPLVKIAPEYNLTLDPSTGMIGAALGREVIILSMDEINEQIAALEATADDLINSLDP    60
SCM58300.1      M.wolf     MEMLPIVKIVPEYNLTLDPSTGMIGASLGREVIILSMDEINEQIAALEATADDLINSLDP    60
CAB41641.1      M.bark     ---MSMIRIAPELNLVMDPETGTITQE-RKDSIQYSMEPVFERVDKLDAIADDLVNSLSP    56
sp|P80655.4|    M.maze     ---MSIVRIAPEINLVMDTESGTVTQE-RKDSIQYSMEPVFERVDKLDAIADDLVNSLSP    56
sp|Q8TU02.1|    M.acet     ---MSMIRIAPEVHLVMDPDTAVVAEE-REDSILYSMDPVFERMDKLDGIAEDLLNSLSP    56
                                 : :  :  :      . :           .   : :.:  *    .     :.

WP_018153518.1             RCSPLKAYPGREGTYKIGGMFQGMFFGFWVTLAILVLVTILV-IKTNLS---LIGL  108
AVB76654.1                 RNTPMKAYAGREGTYKLAGMFQGMFFGFWVTMAVLVLVTILA-VKMNLS---LIGL  108
sp|Q58260.1|               RYPPLNSFPNRDGVYAISGYFKSAFFGFWIGLGIMALLAIILGVKF----------  103
WP_013867106.1             RYKPLKSYDNREGVYCIAGYFKPMFFGVWITLGIGAILALLMGIKF----------  102
WP_048145368.1             EGILASSFPGREQTLKIAGGISAFWYGIAVGLFIAGIIAFELVKVK----------  97
QXO93994.1                 EGSFLESFPNREKSLVFAGGITAMFYGLAVGLLVAGIIVLALM-------------  94
CAA74770.1                 STTTWGTLPGREGVHETAGFLTNFAHGFVIGTMIVALVAFTLAAVYKLHALRLLGL  112
ADL59123.1                 TTIPEGSYPGREGVYLTAGKLTNIVYGFILGLIILFALL----------------  100
SCM58300.1                 TTVSEGSYPGREGVYLTAGKLTNMVYGFILGLIIMFAVLL----------------  100
CAB41641.1                 SNPLLNSWPGRENTSYMAGFYGNTFYGVIIGLAFSGLLALVIYIASLMRGVV----  108
sp|P80655.4|               SKPLLNTWPGRENTSYIAGIYSNSFYGIIVGLAFSGLLALIIYITRLMGGVV----  108
sp|Q8TU02.1|               SKPLLNSWPGRENTSYMAGIYGNAFYGIVVGLAFSGLLALIIFIQRLIEGGM----  108
                                 :  .*:     .*      .*. :   .   : :
```

C

```
WP_012618916.1   M.palu    --------------MTAQITVTEGGIPHNKVMIMGLVGSLIAIYLTYLNTLMGTQAFSFF    46
QXO93995.1       M.hung    --------------MTAKMEASAGAISENTLMIYGIVVALVGTYLTYLNVVTGIAVFSFF    46
CAA74769.1       M.kand    MILRTLLISAVAPGGEEEEVEVAVAISPLKLMTAGLICGILGTAFAWVH--------PLI    52
WP_010870363.1   M.jann    --------MSHGGGGHAA-----ELYPEEQIFAVGIALSLVGCYLANFLSPYG--LGMLI    45
WP_013867107.1   M.okin    --------MSHGGGGHAA-----ELYPENQVLIAGVVLSIIGMYISQYVPQ----ISMLI    43
WP_018153519.1   M.ther    --------MSHGGGGHAA-----ELYPEEQVLAIGAIVSLVGIYIAHMVPQ----VAMLI    43
WP_146778460.1   M.mari    --------MSHGGGGHAA-----ELFPEDQVLAIGAVLSIIGMYVAQFVPS----LAMLI    43
WP_013296334.1   M.marb    --------MSVAAGGPAG-----AAIPESRLMALGILGGLAGIYASAVNPV----IGPVL    43
SCM58302.1       M.wolf    --------MSVAAGGPAG-----AAIPETNLMALGIIGGLAGIYLSAFNPV----IGPVL    43
sp|Q8TU01.1|     M.acet    -------MSAGGAGGEAK-----GGFPPQTIMAIGAIGGLAGIYLGNFMPA----QFSFF    44
CAB41640.1       M.bark    -------MSAGGAGGEAK-----GGYPPQTIMALGIVGGLVGIYLGNFAPP----AYSFF    44
QIB92458.1       M.maze    -------MSAGGAGGEAK-----GAYPQQTLMALGIVGGLVGIYLGHFMPP----AYSFF    44
                                                         ::  *   .: .                .:

WP_012618916.1             GGFGAIAALIWGSSTIKRLCSYGIGTGVPSAGMLAFGSGVIGMLMAT-------KFGMLT    99
QXO93995.1                 GGIGAIAAIWWGSDTIKHLCSYGLGTGVPSAGMVAFGAGAIAMIAGT-------KFGMAS    99
CAA74769.1                 PALAVIPVVVWGADAVRRVAGYGLGTGVPSIGFMGLGGGSVAAILAALS-GNTVPAWAA   111
WP_010870363.1             GGLLASAACVAGANTVRKVAAYGLGTGVPSIGMVSLGMGTLAAVAGVLIPDYFNLPYLVA   105
WP_013867107.1             GGLLAVAATVAGANVTRKVAAYGLGTGVPSIGMVSLGMGTISAIAGVLLPKAFGIPNVTI   103
WP_018153519.1             GGLLAAAACVAGANTTRKVAAYGLGTGVPSIGMVSLGMGTISALAGVLLPPAIGITELAT   103
WP_146778460.1             GGLLAAGACVAGANTTRRVAAYGLGTGVPSIGMVSLGMGTISALAGVLIPSAFGLPVLAT   103
WP_013296334.1             ASLGAVCAIVWGADAIRRVASYGLGTGVPSIGYMSVSIGVGVVAGL--ASVFVVPAIAV   101
SCM58302.1                 ASLGAVCAIVWGADAIRRVASYGLGTGVPSIGYMSLAIGVGAVAGL--ASVFVVPAIAV   101
sp|Q8TU01.1|               GGLGAICAMVWGADAVRRVASYGLGTGVPSIGMISLGMGIVAALFGL--S----VGGIAG   98
CAB41640.1                 GGLGAICATDWGADAVRRVASYGLGTGVPSIGMLALGMGILAALFGL--S----VGGTAG   98
QIB92458.1                 GGIGAICATVWGADAVRRVASYGLGTGVPSIGMLALGMGILAALFGL--A----LGGIAG   98
                            .: .  .   *:.. :::..**:****** * :... * :. : .

WP_012618916.1             PVLALIIAAIVGAILGFISNNILNMRIPVMIQSLTELAAVGALVLLGFSAMATGGFSMAT   159
QXO93995.1                 PIVTLILAAIIGAVIGYIANNIINMNIPVMIFSLMKLSIVGALTMLGFAAMCTGTFMFNG   159
CAA74769.1                 AIIGTVIGAVVGALLGVLDRRVIKMKIPVMERCSTEIVASGTLALICLMAAVAGDFTWSA   171
WP_010870363.1             PIITLIVSAVIGYIVGRLTVNPVGMKIPIMVRSMTFLSIAGAMALLGFTVAYVGSLEPQK   165
WP_013867107.1             PIVAAVVAVILGLIVGKLTVKPIGMKIPIMVQSMTKLSLMGTLSILGFCSAYAGGFSPDI   163
WP_018153519.1             PIVTAATAIVIGFIVGKLTVNPVGMKIPIMVQSMTKLSLMGALAILGFCTAFAGGFSADL   163
WP_146778460.1             PILAAVIAVVVGFIVGKLTQNPVGMKVPIIVSSMTKLSLMGALAILGFCTAFAGGFSADL   163
WP_013296334.1             PVVALILAMILGVVVAVLGKKIVKMKIPILEKCTAEISGAAALSVLGFSAAIAGSYTLQT   161
SCM58302.1                 PVVGFVLAMILGIVVAVLGKRIVKMKIPILEKCTAEISGAAALSVLGFSAAIAGSYTMQA   161
sp|Q8TU01.1|               PIVSFIAAAIIGAVIGVLANKVIGMGIPIMEQAMVEIAGAGTLVIIGLSVVIAGTFDYAE   158
CAB41640.1                 PIVAIVVAAIIGGVIGALANKVIGMGIPIMEKAMVEISCAGTLVILGLSVVIAGSFDFAS   158
QIB92458.1                 PILAVVVAAIIGGVIGALANKVIGMGIPIMEQAMIEISCAGTLVILGLSVVIAGSFDYAA   158
                            ::    . ::* ::. :  . : * :*::  .   :   .:: :: :    .*
```

```
WP_012618916.1      LTTAT----------------TTVLGTQVASYTASLIGGSILAVLFMLGAIALQHGFNAC   203
QXO93995.1          LVIGGMTLSMEPAAEAAAGGAQTFMVTVLPEFAGSLIGGAALAVIFFLGAMALQHPFNAC   219
CAA74769.1          VYSK-------------------------------VIATGLIAVLWAICAISLLHPFNAC   200
WP_010870363.1      YIDY-------------------------------ALNNGMMALAFIAAGMAILHPFNAC   194
WP_013867107.1      IIPG-------------------------------AIKNGIIALAFIAPGMAILHPFNAC   192
WP_018153519.1      IING-------------------------------AVNNGIIGLAFITAGISILHPFNAC   192
WP_146778460.1      IING-------------------------------AINNGVIALAFIAAGMSILHPFNAC   192
WP_013296334.1      MLTS-------------------------------VITTGFIGLLFILNTMAIQHPFNAC   190
SCM58302.1          MLTS-------------------------------VISTGFIGLLFILNTMAIQHPFNAC   190
sp|Q8TU01.1|        VVEY-------------------------------VVANGYIALIFIIGGMGILHPFNAN   187
CAB41640.1          VVQY-------------------------------VVANGYIALIFIIGGMGILHPFNAS   187
QIB92458.1          IIEN-------------------------------VIANGYIALIFIIGGMGILHPFNAC   187
                                                        :  . :.: :    :.: * ***

WP_012618916.1      LGPNEKQDRTLMLTAECGFLSMIMVAVISFAFIGIGAA------VLSLLISLVGWYYTYT   257
QXO93995.1          LGPNESQDRTLMLAIEVGFLSMFVVAVMSFAFLDLLSA------TVGLIISLIGWIYTYK   273
CAA74769.1          LGPSETQERTLWLGAECGSLCTVVAGLATA-------------NPVVLLAGAAAWLITFW   247
WP_010870363.1      LGPNESHKRTLTLAVACGFITWFVFSVVKL-------------DIVSIIVSIILWAIVYV   241
WP_013867107.1      LGPNESHKRTFINAIACGLLTWFIFSMAKL-------------DAISIVVSLILFAIAYY   239
WP_018153519.1      LGPNESHKRTLMLAIACGLLTWFIFSIAKL-------------DVISIVVSGIFWVVSYS   239
WP_146778460.1      IGPDESHKRTITLAVACGLMAWLIFSIAKL-------------DIVSTAVAAIFWLYTYG   239
WP_013296334.1      LGPNENQTRTLKLAASTGFISMAIVG--------LLGIGLNPSWWLVSLIGALCWIVAFR   242
SCM58302.1          LGPNENQTRTLKLAASTGFLSMAIVG--------LLGIGLNPGWWLVSLVGALAWVVAIK   242
sp|Q8TU01.1|        LGPDEKQDRTLSVAVEKAAIALIITGFASSLHEGLMAAGLN------IAVGVIIWAWAFM   241
CAB41640.1          LGPDEKQDRTLMLAVEKGAIALIIAGFASSLHEGLMAAGLN------MLIGIIIWYVAFS   241
QIB92458.1          LGPDESQDRTLILAVEKAAIALIITGFASSLHEGLMTAGIN------ILVGLVIWYVAFS   241
                    :**.*.: **:      . :   : .                        .   :

WP_012618916.1      QFFALSKRDAASWLDAKPILEVESEA   283
QXO93995.1          QYIALSKRDAYAWLDSKPIIEVGGDQ   299
CAA74769.1          KFWELTKRDAADVVWTGIVPKGE---   270
WP_010870363.1      KFVKMSFKDACAVLHVPEIPKKEE--   265
WP_013867107.1      KFVKQSLNDACCVLYTPELPKKEE--   263
WP_018153519.1      LFVKQSLNDACDVLYTPELPKKEM--   263
WP_146778460.1      SFVKMSLADACEVKYVPELPKKE---   262
WP_013296334.1      AFVSASFEEAASVKWSGLWPKEEEH-   267
SCM58302.1          SFIEASFEEAASVKWSGLWPKEEEQ-   267
sp|Q8TU01.1|        KYYGYVKRDSYAVVGTGLLPSAEELE   267
CAB41640.1          KHYALIKRDAYAVVGSGMLPSSEELQ   267
QIB92458.1          KYYALIKRDAYAVVGTGLLPSAEELQ   267
                     .      ::          .
```

D

```
WP_012618915.1    M.palu    MTAIAAK-GGPASMDMPSTLIGLVLFIILLGLMFVLGP-ISAFYALIGIVVGGVLIGFGV   58
QXO93996.1        M.hung    MSALGGKAAGGEGINPTGSVVGIVILLVSIAATYALG---FSIVPLIGIIIGGALIGFGV   57
WP_010870362.1    M.jann    -----------------------------------MDI-VSAIVPLIEMTIAGAIINASV   24
WP_011977193.1    M.mari    -----------------------------------MDA-TSFILPLAEITIAGAIINASV   24
WP_018153520.1    M.ther    -----------------------------------MDA-TSLIIPLAEITIAGAVINASV   24
WP_013867108.1    M.okin    -----------------------------------MDA-VSLIIPIAEITVAGAIINASV   24
sp|Q8TU00.1       M.acet    ----------------------------------MIDALMANILWLVFIIIGGVLISWSV   26
CAB41639.1        M.bark    ----------------------------------MIDAILGNIIWMALITIGGVLISWSV   26
BBL65799.1        M.maze    ----------------------------------MIDAILGNILWMAFIVIGGVLISWGV   26
ADL59125.1        M.marb    -----------------------------------MDP---LL-LIGAITAGGVLIGGGV   21
SCM58303.1        M.wolf    -----------------------------------MDP---LL-LIGAITAGGVLIGGGV   21
CAA74768.1        M.kand    -----------------------------------MDK---LIAVLVLITLGSIMVNVGV   22
                                                               :.     :  :  :  .. ::. .*

WP_012618915.1              HFVPVGGAPAAMGQAPGIATGVAMLAAGAGLAGLFGGAWAAEQ-------GTT--IAVIT   109
QXO93996.1                  HFVPVGGAPAAMGQAPGIATGVAMLAAGAGLAGLFGGAWAYEA-------TGDFAVAVAG   110
WP_010870362.1              HFIPVGGAPAAMATSTGVGTGTTQLAAGAGFTGLMGAAVMASNVGLS---PIGMALIMIS   81
WP_011977193.1              HFVPVGGAPAAMATSTGVGTGTTQLAAGAGFTGLLAAATMASQAGVSLANPVHMLLIMLS   84
WP_018153520.1              HFVPVGGAPAAMATSTGVGTGTTQLAAGAGFTGLMAAAAMASQAGISLANPVHLLMIILS   84
WP_013867108.1              HFVPVGGAPAAMATSTGVGTGTTQLAAGAGFTGLMAAAVMAAQSGISLSNPVHLTLILLS   84
sp|Q8TU00.1|                HFVPVGGAPAAMAQATGIGTGTVQLAAGAGLTGLVSAGFMMNV-------TDNLPLILAS   79
CAB41639.1                  HFVPVGGAPAAMAQATGIGTGTVQLAAGAGLTGLVSAGFMMNV-------TDNLPLILAS   79
BBL65799.1                  HFVPVGGAPAAMAQATGVGTGTVQLATGAGLTGLVSAGFMMNV-------TDNFPLIVAS   79
ADL59125.1                  HFVPVGGAPAAMATATGVGTGTAMLAAGAGLTGLITAAAMTG---------QSPLMIMAA   72
SCM58303.1                  HFVPVGGAPAAMATATGVGTGTAMLAAGAGLTGLITAAAMTG---------QPPLLIMAA   72
CAA74768.1                  HYVPVGGAPAAMATATGVGTGTTQLAAGSGLTGLITAAAMSQ---------KPFLVILWN   73
                            *::*********. : *:.**.. **:*:*::**. ..                 : :
```

```
WP_012618915.1      GGVGGALMMAITCLMVNAIYVYGMGIPAASGKVAKDPLTGDTQAAYKSQGTEGHGLPFIS   169
QXO93996.1          GAVGGGLMMAITCLMVNATYVFAMGIPAASGKVAKDPITGDTFPEYKSQGTEGHGLPFIS   170
WP_010870362.1      GAVSSMIMLGVTMLIGQLIYVFGVGVVPAADKCEIDPITKDPQKPYVTPGTTGHGVPTVC   141
WP_011977193.1      GAVGAMIMLGLTMLIGQIIYVYGIGIVPAADKCEKDPITGDIQKPYITPGTTGHGIPTVC   144
WP_018153520.1      GAVGSMIMLALTMLIGQLIYVFGVGVVPAADKCEKDPITGDYQKSYITPGTTGHAIPTVC   144
WP_013867108.1      GAVGSMIMLGLTMLIGQLIYVYGVGVVPAADKCDKDPITGDYQKSYITPGTTGHGIPTVC   144
sp|Q8TU00.1|        GAVGAMIMISVTMIVGTWVYVYGVGCVPSSAKVKYDPITKYRQDLYVSQGTEGHGLPTVS   139
CAB41639.1          GSVGAMIMIAVTMIVGSIVYVYGVGVVPSSAKVKVDPITKYRQDLYVSQGTEGHGLPTVS   139
BBL65799.1          GAVGAMIMIAVTMIVGTWIYVYGVGCVPSSAKVKVDPITKYRQDLYVSQGTEGHGIPTVS   139
ADL59125.1          GAVGSMLMIGITMLVGNLIYVFGVGTVPVSAKVSVDPITGMEQEKYVTPGTEGHGLPTVC   132
SCM58303.1          GAVGSMLMIGITMLVGNLIYVFGVGTIPVSAKVAVDPITKMEQEKYVTPGTEGHGLPTVC   132
CAA74768.1          GALGAATMMAITMLVGNFIYVYGVGCPPCSAKVDKDPITGWDQEAYVTPGTEGHGIPTVS   133
                    *.:..  *:.:* ::    **:.:*     : *  **:*       * : ** **.:* :.

WP_012618915.1      YVGGVIGGFFGGAGGTLIYLELLGVYEQALPTLFNAKLEQIMPLAVSLAGIFAVGMFLVN   229
QXO93996.1          FFGGVVGGFIAGLGGTLIYIELLDVYHAGLPAIMQASPEAIEPLAVSLAGIFAIGFFLVN   230
WP_010870362.1      FVSGLIGAALGGIGGALAYIALRKLGLD-----------------PGVAGMLAVGFFFIN   184
WP_011977193.1      FVSGSIGAALGGLGGALAYIALQQLGFA-----------------AAIAGVLAVGFFFMN   187
WP_018153520.1      FVSGLIGAAFGGLGGALAYIALKQLGFS-----------------SEVAGVIAIGFFFMN   187
WP_013867108.1      FISGLIGAALGGIGGGLAYVAFKLLGFS-----------------SEVAGIIAVGFFFMN   187
sp|Q8TU00.1|        FVSGVIGGLLGGIGGALVYYSLIEVGLTAGLS-TGTSSGVTGHELVGIAAMFAIGIFFVN   198
CAB41639.1          YVSGIIGGGLGGIGGSLVYYSLIEVGMSAGLEAVGVTNSVTGHELVAVAAIFAIGIFFVN   199
BBL65799.1          FVSGVIGAALGGIGGSLIYYSLIEVGVSVGLERVGVTSAVTGNSLVAVAAIFAIGIFLVN   199
ADL59125.1          FVSGIIGGALGGIGGGLIYWALNEALKTLSYGAM---------GAAGVAAIFAVGIFFIN   183
SCM58303.1          FVSGIIGGALGGIGGGLIYWALNEALKTLSYGAM---------GAAGVAAIFAVGIFFIN   183
CAA74768.1          FVSGILGGLLGGSGGAMVYYALYKVLGM----------------SAALAGILAMGFFYAN   177
                    :..* :*. :.* ** : *  :                          :*.::*:*:*  *

WP_012618915.1      SVLAAYNITGTIEGPHDPKFKRFPRAIVASMIASALCGLVAILLVVMGKF----   279
QXO93996.1          AVLAAYNITGTIEGPHDPKFKRVPRAVIGCAVASAFCGLISMLIVLNTGM----   280
WP_010870362.1      AVLASYNIGGTIEGFHDPKFKKMPNGVIASTVASLLFGIISVLMV----L----   230
WP_011977193.1      AVLASYNIGGTIEGFHDPKFKKMPNGVIASFVSSLIAGAVLIGMA----MGL--   235
WP_018153520.1      AVLASYNIGGTIEGFHDPNFKKVPNGIIASFISSLVTGAILAGMA----LGF--   235
WP_013867108.1      AVLASYNIGGTIEGFHDPKFKKIPNGIIASTVGSVIAGIVIAGMS----LGI--   235
sp|Q8TU00.1|        AVIPSYNIGGTIEGFHDPKWKKWPKAVISSFVATILCAIVAVIA--ISQLGGI-   249
CAB41639.1          AVIPSYNIGGTIEGFHDPKFKKWPKAVVSSLVASIMCAIVAVVA--IAQLGGI-   250
BBL65799.1          AVIPSYNIGGTIEGFHDPKFKKWPKAVISSLVASILCAIVAVIA--IAQLGGI-   250
ADL59125.1          AVIASYNIGGTIEGFHDPKFKRIGRGIVACLIASIVAGAL----STLLVYGGVF   233
SCM58303.1          AVIASYNIGGTIEGFHDPKFKRIGRGIVACLIASIVAGAL----STLLVYGGVF   233
CAA74768.1          AVLASYNIGGTIEGYHDPKFTRLPKAVVCSLVFGIVASVIAYYLSTLM------   225
                    :*: :*** ***** ***::.:  ..:: . :   . . :
```

E

```
WP_012618914.1 M.palu    MVDNIVFGIGITALAGALAAVAGAAEDVESDIGSQGDPNSQVQLAPQMGFIHRIFNKAVA   60
QXO93997.1     M.hung    -MEEIVLGIGITALAGAFATVAGAAEDTESDIGSQGDPNSQVQLAPQMGYIHRIFNKAIS   59
CAA74774.1     M.kand    --MVGFTEIGLAAAMGALATIAGAFEDAESDVGSQSNPNSQVQLAPQMMNFHRYFNKAIS   58
sp|Q8TTZ9.1|   M.acet    --MEPLIGMGVLALIGVAATIAGASEDLESDVGSQSNPNSQVQLAPQMMFPHRIFNKAVS   58
CAB41638.1     M.bark    --MEPLISMGVLALIGVAATIAGASEDLESDIGSQSNPNSQVQLAPQMMFPHRIFNKAIS   58
WP_243466678.1 M.maze    -------MVTGFSFYGVAATIAGASEDLESDIGSQSNPNSQVQLAPQMMFPHRIFNKAIS   53
ADL59126.1     M.marb    -MDPMITGLGVVALMGAAATIAGAAEDLESDVGSQSNPNSQVQLAPQMGHLHRIINKAVS   59
SCM58305.1     M wolf    -MDPMITGLGVVALMGAAATIAGAAEDLESDVGSQSNPNSQVQLAPQMGHLHRIINKAVS   59
WP_018153521.1 M.mari    -MDPTLITLGALALAGAATTVAGCAEDLESDVGSQSNPNSQVQLGPQMGNIHRYFNKAIS   59
WP_181504881.1 M.ther    -MDPTLISLGALALAGAAATVSGCAEDLESDVGSQSNPNSQVQLGPQMGNIHRYFNKAIS   59
WP_010870361.1 M.jann    -MDATLIALGALALSGALATVAGCAEDLESDVGSQSNPNSQVQLAPQMGNIHRYFNKAIS   59
WP_013867109.1 M.okin    -MDPTIITLGALALAGAAATVSGCAEDLESDVGSQSNPNSQVQLGPQMGNIHRYFNKAIS   59
                                 :   :  *. ::::*. ** ***:***.:*******.***   ** :***::

WP_012618914.1            GEPPAYGLWCALGAGIAWAFMAM-QFNPVLAIILGCALATFVQGVYATTAYLGRTASLA-   118
QXO93997.1                GEPPAYGAWCAIGAGVAWALMQI-NYNPALAIILGSVIAVFVQGIYCTSGYLGRIASLA-   117
CAA74774.1                GEPVSYMLYGAIAGTVTWVMMTKFGLPFLAAAAVGVGVNALIHMVFATTAHLGRMASAA-   117
sp|Q8TTZ9.1|MTRE_METAC    GEPPSNALMCSVGAAVATVLISEFTLSPLFALVLGALIAACVHGTFAVTSTMGRAASQS-   117
CAB41638.1                GEPPSNALMCSIGAAVATVLISEFTMSPLFALVFGSLIAACVHATFAVTSTMGRCASQS-   117
WP_243466678.1            GEPPSNALMCSIGAAIATVLISEFTVSPLFALVFGSVIAASVHATFAVTATMGRCASQS-   112
ADL59126.1                GEPVAYGTWCGIAGSVAFVLMNSMQLPVIMAIAIGAVIAAMVHTTYAVTSHMGRIVSQS-   118
SCM58305.1                GEPVAYGTWCGIAGSVAYVLLNSMQLPVIMAIAVGSVIAAMVHTTYAVTSHMGRIVSQS-   118
WP_018153521.1            GEPVSYGLYVAVAGAVAWALMST-NLNAILALVVGSGVAALVHGAYSVSSYLGRIVGQSK   118
WP_181504881.1            GEPVSYGLYVAVAGTIAWALINA-GLNVVLAIIVGAGVAAIVHGAYSVSAFLGRTVGQSK   118
WP_010870361.1            GEPVSYGLYVAVAGTVAYAIMQM-GLNPILALILGAGVAAFVHGAYAISAYLGRIVGQSK   118
WP_013867109.1            GEPVAYGLYVAVAGSVAWAIMHI-GLNPILALIIGAGVAAFVHGAYAVSAYLGRIVGQSK   118
                          *** :     .:.. :: .::         *  .*  : . ::  :. :. :** .. :
```

```
WP_012618914.1             KFGQPVYIDILKSMTSVTMAHAFVAVFTTVSLCYLMN---AALGHPFPLPLLGLVWGIAL      175
QXO93997.1                 KFQQPVYIDIIKSLLSVTMAHAFVAIFCTVAMCYLMA---SALHHPFALPLLGLVWGIAL      174
CAA74774.1                 EFGHPIYLDVVLSHLGPIAGFGGIATFAIVSLAYIQW---ALLKHPFPLPLLAALWGVTV      174
sp|Q8TTZ9.1|MTRE_METAC     RFKQPVYLDMIRSHTPVIMGYSFITTFCILVVSYLMT---VVLGHPFPLTMLAFIWGITV      174
CAB41638.1                 RFKQPIYLDMIRSHITPIMGYAFITTFCILVVSYLMT---VVLGHPFPLTMLAFIWGITI      174
WP_243466678.1             RFKQPIYLDMIRSHTPAIMGYAFITTFCVLIVSYLMT---VVLGHPFPLTMLAFIWGITI      169
ADL59126.1                 QFNQPLFMDMLVQHLGPIAGHGFIVTFCTVGLSYLMTLPIPGFAHPFPLPLLAVLWGITI      178
SCM58305.1                 QFNQPLFMDMLVQHLGPIAGHGFIVTFCIVGLSYLMTLPIPGFGHPFPLPLLAVLWGITI      178
WP_018153521.1             NFGQPVYMDVLITHLGPIVGHGFIAIFCMALAAYLAS---TALGNPFPLPLIALIFGITV      175
WP_181504881.1             KFGQPVYMDVLTSHIGPIVGHGFIAVFTMTLAAYLAT---TALGNPFPLPLVSLIFGITV      175
WP_010870361.1             NFGQPVYWDVVMSHLGPIVGHGFIAVFCMVLMAYLAN---TILGNPFPLPLIALIFGITV      175
WP_013867109.1             NFGQPVYMDVILSHVGPIVGHGFIAVFCMLFVAYLAT---TILGNPFPLPLVAFIFGITV      175
                           .* :*:: *::         ... :. *     .*:         : :** * ::. ::*:::

WP_012618914.1             GAAGSATGNPFYGKERQYQNQKFGAGVPISASGNIVRYAEAGQRNSLDNGWFSSKLGGPA      235
QXO93997.1                 GAAGSATGNPFYGKERQYQNQKYGAGVPISASGNIVRYAEAGQRSSLDNGWFTTKFAGPA      234
CAA74774.1                 GAIGSSTGDVHYGAERLYQHYPFGGGVPVAAHGNITRKAETGIRNSMDSVYFCAKFGNPL      234
sp|Q8TTZ9.1|MTRE_METAC     GAIGSSTGDVHYGAEREFQQFEFGSGLNASNSGNIVRYGESGVRNGYDNSWFCAKFGGPV      234
CAB41638.1                 GAIGSSTGDVHYGAEREFQQFEFGSGLNASNSGNIVRYAESGLRDGFDNSWFCAKFGGPV      234
WP_243466678.1             GAIGSSTGDVHYGAEREFQQFEFGSGLNASNSGNIVRYAESGLRNGFDNSWFCAKFGGPT      229
ADL59126.1                 GAIGSSTGDVHYGAEREYQQYPFGGGIPVAIHGDITTKAELGARNSMDVVHFCAKYGGPL      238
SCM58305.1                 GAIGSSTGDIHYGAEREYQQYPFGGGIPVAIHGDITTKAELGARNSMDVAHFCAKYGGPL      238
WP_018153521.1             GAIGSSTGDVHYGAEREYQKYLFGGGIPVANQGDIDIKAEYGIRNGLDSSYFCSRLGGPL      235
WP_181504881.1             GAIGSSTGDVHYGAEREYQKYPFGGGIPVANQGDIDIYAEYGIRNGLDSSYFCSRFGGPL      235
WP_010870361.1             GAIGSSTGDVHYGAEREYQKYPFGGGVPVANHGDIDIKAEYGLRNGMDSSYFCSRLGGVL      235
WP_013867109.1             GAIGSSTGDVHYGAEREYQKYPFGGGIPVANQGDIDIKAEYGLRNGIDSSYFCSKLGGPL      235
                           ** **:**: .** ** :*:  :*.*:  :  *:*    .* * *.. *   * :: ..

WP_012618914.1             SGICFGLIVFLELWRTVIFEKVGG---------GWGSIIVGVIMILIFAIVDRYIETWAR      286
QXO93997.1                 SGICFGLIVFLELWRTVLFEKMMN---------GWGAIIMGVVLILVFTFIDRWVEVWGR      285
CAA74774.1                 TGLCFGLVVFFSTWAGLFGQWGAV-------------IAMGLV-TLGCLIVSNRVEKKAR      280
sp|Q8TTZ9.1|MTRE_METAC     TGMAFGMTVFLGSWVTTVFDPAVS------ISRGWISVVAGVIIVLILIFWNWKIEVKAR      288
CAB41638.1                 TGLAFGMTVFLGSWITTIFDPAKG------L--GWLSVIAGIVIVFILIIWNWKMEVYAR      286
WP_243466678.1             TGIAFGMTVFLGSWITTIFDPAKG------LSMGWLSVVAGVIIVLILIIWNWKIEVQAR      283
ADL59126.1                 TGFAFGAIVFLSFWNTIVFGITGG-------------IISGLIIVLLLIILNNRLEVFAR      285
SCM58305.1                 TGFAFGAIVFLSFWNTIVFGITGG-------------IISGLIIVLLLIILNNRLEVFAR      285
WP_018153521.1             TGLCFGLIIFLDGWRGIVGNIVG----SDLITKSVVAILVGLLIVGVAAYANRKIEVYAR      291
WP_181504881.1             TGLCFGLIIFLDGWRSILGNILG----GDLVTKTSIALLVGLLVVAVAAVINRKLEVYAR      291
WP_010870361.1             TGLCFGLIVFLDGWRGVLGDILKGGQGGSVITASIISIVIGLIIVAILAIINRKVEVFAR      295
WP_013867109.1             TGLCFGGIVFLDGWRGLMGDILG----GDLVTKSSIAIVIGIIFVAIAAYINRVIEVYAR      291
                           :*:.**  :*:  *   .                    :  *::        .  :*  .*
WP_012618914.1             KNYGPYTTEETSA-----  299
QXO93997.1                 KTYGPYTTEEASS-----  298
CAA74774.1                 ESYGTYEDVEMDEICDPV  298
sp|Q8TTZ9.1|MTRE_METAC     NAYGPYKEDKTEEASA--  304
CAB41638.1                 KAYGPYKEDKTEEASA--  302
WP_243466678.1             KAFGPYKEDKAEEASA--  299
ADL59126.1                 NRYGPYKEEE--------  295
SCM58305.1                 NTYGPYKEDE--------  295
WP_018153521.1             NKYGPYTNR---------  300
WP_181504881.1             NKYGPYRN----------  299
WP_010870361.1             NKYGPYTK----------  303
WP_013867109.1             KKYGPYTDR---------  300
                           : :* *
```

F

```
WP_018153668.1  M.ther     KIAQAIKDCISKDPGAIDEDAVVLDLEGGGGAGEGGDEEGLSIEGIPTVAEPDLE---YT       57
sp|Q58262.1|    M.jann     -------------------------------------------MGVEVSNKPNVS---SI       14
AEH06920.1      M.okin     -------------------------------------------MSLEISNKPNTK---SI       14
WP_011019853.1  M.kand     ------------------------------------MAEEGSELKEVIIGAPAMADTDRA       24
SCM58296.1      M.wolf     -------------------------------------------M-IILSNKPNIR---GI       13
ADL59121.1      M.marb     -------------------------------------------M-IILSNKPNIR---GI       13
CAB41643.1      M.bark     ----------------------------------MKVAEEYDKG-VPMMLAPQMG---AI       22
KKI06210.1      M.maze     ------------------------------------MAEEHEKG-VPMVLAPQMG---AI       20
sp|Q8TU04.1|    M.acet     ----------------------------------MRMAEEYGKG-VPMVLNPQMG---AI       22
WP_012618919.1  M.palu     ------------------------------------MAEEGSK----AAGPIRMV---AI       17
QXO93992.1      M.hung     --------------------------------------MSDEK----KSGPIRMA---AI       15

WP_018153668.1             KKLLDSLEYKVGLITRDLGLASGVQSQAVEGALLGSLFAVVLIGIPIILKLLLG-    111
sp|Q58262.1|               QSYVEDLEYKVGLITRNRGLESGTESAGTKGLIIGVVSAIVLMGIPLALYFLMK-    68
AEH06920.1                 ESIMDKLEYKVGLITRNRGLESGIESSSVKGLAIGVIFGIVLLAIPIIIRLYQ--    67
WP_011019853.1             DTYVNDVRDSSQFFGRDARLYFGLNVNRFAGLACGMVFAGVLLVPLLLLA-F---    75
SCM58296.1                 KNVVEDIKYRNQLIGRDGRLFAGLIATRISGIAIGFLLAALLVGVPAMMSMLGVI    68
ADL59121.1                 KNVVEDIKYRNQLIGRDGRLFAGLIATRISGIAIGFLLAVLLVGVPAMMSILGVI    68
CAB41643.1                 DATVESIRYRAQLIARNQKLDSGVAATGMIGFAAGFLFSLLMVIVLPLL--FW--    73
KKI06210.1                 DATVESIRYRAQLIARNQKLDSGVAATGIIGFAAGFLFSLLMVIVLPVA--VGL-    72
sp|Q8TU04.1|               DATVESIRYRAQLIARNQKLDSGVMSTGIIGFAAGFLFSLLMVIILPLM--AGL-    74
WP_012618919.1             NNMVENMRYKSQILARTNKLESGIMDSGLVGFAAGMLVALVLIVVPALVLM----    68
QXO93992.1                 DTMVADMKYKGQILARTNKLESGIMGSGIVGFAIGLAFALILVV-P-VILMGGV-    67
                           .  : .:.    :: *   *  *       *   *   . :::
```

G

```
WP_018153669.1  M.ther      -----MSEIPTVVTPSKDFKKLQEKLSEIDETVENTNAEIVQRLGKKAGRDVGIVYGFII     55
WP_011867789.1  M.mari      -----MSEIPTVVTPTKDYKKLQAKLDEIENTVENTNAEIIQRTGKKAGRDVGIAYGLAI     55
ADL59120.1      M.marb      MSEEEKTTIPRVLVSADEFNKANEKLDEIEEKVEFTVGEYSQRIGQQIGRDIGILYGIVI     60
SCM58295.1      M.wolf      MSDEEKTTIPRVLVSADEFNRANERLDEIEEKVEFTVGEYSQRIGQQIGRDIGILYGIVI     60
sp|Q8TU05.1|    M.acet      ----MDGKAPAAYVDPAEFNEVMKRLEKIDEKVEFVNSEVAQRIGKKVGRDIGILYGAVV     56
CAB41644.1      M.bark      ----MDGKAPAAFVEPGEFNEVMKRLDKIDEKIEFVNSEVAQKIGKKVGRDIGILYGGFI     56
QIB92462.1      M.maze      ----MDGKAPAAFVEPGEFNEVMKRLDQIDEKVEFVNSEVAQRIGKKVGRDIGILYGGVI     56
WP_010870367.1  M.jann      --MSEDEKLPQVIMDPADYEALKKRLDELEKKVENTNAELFQLAGKKVGRDIGILYGLVI     58
WP_013867103.1  M.okin      MGEDEKTPVPQVIMDPKDYEALMQRLDELETKVENTNAEVYQTAGRKVGRDTGILYGLTI     60
                                     * .     :::    :*.::: .:* . .*  *  *:: *** ** **  :

WP_018153669.1              GFVIVVVLGKILPLFQFIK------- 74
WP_011867789.1              GFIFVYVLGTVLPLFDLIK------- 74
ADL59120.1                  GLIILAVTNILFAGLLKGLLKSLFGL 86
SCM58295.1                  GLIILAVTNILFAGLLRGLLRSLFGL 86
sp|Q8TU05.1|                GLLLFLIYVSVSSMFTI--------- 73
CAB41644.1                  GLLLFLIYTVVSSMFM---------- 72
QIB92462.1                  GLLLFLIYVQISSMFM---------- 72
WP_010870367.1              GIILSYILPALIKIIQILSLKVLVQQ 84
WP_013867103.1              GLILYNIFPLIIKLIDYLSMLK---- 82
                            *:::  :   :   :
```

H

```
WP_012618921.1 M.palu       ----MFRFEKEQQVWDFNGTKIGGQPGEHPTVLGASIFYNKHEIVLDDHKGTIDKPKAEA     56
QXO93990.1     M.hung       ----MFMFEKEQTVLDFNGYKLGGQPGEYPRALGASIFYNKHETVLDEHTGKIDKAKAEA     56
CAA74773.1     M.kand       MDRCSTTPGKEQKVCDICGVKVGGQPGEYPTVLAGTIFYAGHKIVKDEDKGIFDEEAAEE     60
CAA10790.1     M.bark       ----MFKFDKKQEVFEIGGVKFGGQPGEFPTVLVSTMFYARHKIVTDEDKGIFDRAAAET     56
WP_011033486.1 M.maze       ----MFKFDKKQEVFELGGVKFGGQPGENPTVLVSTMFYARHKIVTDEDKGIFDRAAAET     56
sp|Q8TU06.1    M.acet       ----MFKFDRKQEVYEVGGVKFGGQPGEYPTVLVSTMFYARHKIVTDEDKGIFDRAAAET     56
WP_013296329.1 M.marb       ----MFRFDKEQIVLDIAGTKIGGQPGEYPTVLAGTIFYGGHSIIEDEKAGVFDKDKAEA     56
SCM58293.1     M.wolf       ----MFRFDKEQIVLDIAGTKIGGQPGEYPTVLAGTIFYGGHSIIDDEKAGVFDKDKAEE     56
WP_181487234.1 M.mari       ----MFRFDKEQMVIEFAGAKFGGQPGEYPTALSGTIFYSRHKIVEDAKKGIFDKKAAEA     56
WP_010870368.1 M.jann       ----MFKFDREQMVVEIAGRKIGGQPGEYPTALAGTIFYARHKIVEDERKGIFDKAAAED     56
WP_018153670.1 M.ther       ----MFKFDKEQMVIEIAGRKFGGQPGEYPTGLSGTIFYARHKIVEDERKGIFDKKAAED     56
AEH06918.1     M.okin       ----MWAYDKEQAVFEIAGRKVGGQPGEYPTGLSGTIFYARHKIVEDERKGIFDKAAAEE     56
                                     ::* * :. * *.****** *  * .::**  *. : *   * :*.  **

WP_012618921.1              LWNRCQELTDETGVHFFIQIIGEFGEALESYFDWFASIDDKTAFLMDSSAPAARMQAAKY     116
QXO93990.1                  LWNRCVELYDITGHWYFCQIIAEFGEAFESYIDWFCSIDDQFPFLMDSSAPAALAHACKY     116
CAA74773.1                  LIKMEEELADETGNPMMAHIMGESEEAIIRYLDWVADVTD-APIIVDSTEAEVKVAAVKH     119
CAA10790.1                  LWNTQVSLSDATGNPYVNQIVGETPESIKRYIDWFVEIDDRTPFLIDSSAGNVRAAAAQY     116
WP_011033486.1              LWNTQVSLSDATGLPYVNQIVGETPESIKRYIEWFVGIDDRTPFLIDSSAGNVRAAAAQY     116
sp|Q8TU06.1|                LWNTQVSLSDATGNPYVNQIVGETPESIRKYIDWFIEIDDKTPFLIDSSAGEVRAAAAQY     116
WP_013296329.1              LIKTQEEMSDVTGNPHIVQTFGQTPEAIVKYLEFVGDITD-APFFIDSTSGEARIAGAEY     115
SCM58293.1                  LIKTQEEMSDVTGNPHIVQTFGQTPEAIVKYLEFVGDVTD-APFFIDSTSGEARIAGAQY     115
WP_181487234.1              LINKQAEMQDITGNSAFVQVFGGTEEALVNYIDFVSEVWD-GPMLLDSTSGKARMAAANR     115
WP_010870368.1              LINKQAEMEDITGNPALVQVFGGTPEALVNYIDFVAEVWD-GPMLLDSTSGEARMAAAKR     115
WP_018153670.1              LINKQAEMEDITGNPALVQVFGGNPEALTKYIDFVAEVWD-GPMLLDSTSGEARMAAARR     115
AEH06918.1                  LINIQAEMEDITGNAAFVQIFGGNPEALTKYIDFVAEVWD-GPMLLDSTSGEARMAAARR     115
                            * :   .: * **   . : ..   *::  *:::.  : *   :::**:   .   . .

WP_012618921.1              VTEVGLANRAIYNSINGSISQEEIDVLAKSDVDAAIVLAFNPADPSVAGRQKVLGEGGVA     176
QXO93990.1                  VTDAGIADRAVYNSINGSIGPENIEAIKNSDVDAAIVLAFNPGDPSVRGREKVLAEGGVA     176
CAA74773.1                  GQEVGLAERVVYNSINASVEDEEIQAIKESDCNSAIVLAFNPMDASVEGRMKILTEGEEG     179
CAA10790.1                  CTEIGVADRAIHNSINASIEQEEIDVLTESDVEAAIVLAFNATDPTVKGKMDILEVGGSG     176
WP_011033486.1              CTEIGVADRAIHNSINASIEQSEIDVLTESDVSAAIVLAFNATDPTVKGKIDILEVGGSG     176
sp|Q8TU06.1|                CTEIGVANRAIHNSINASIEQSEIDILTESDVEAAIVLAFNATDPTVKGKLDILEVGGSG     176
WP_013296329.1              ASEVGLEDRAIYNSVNMAADESELEALKNTKLSASIVLGFNPMDPTVEGKIGIWEDGAGT     175
SCM58293.1                  ASEVGLEERAIYNSVNMSADESELEALKDTKLSASIVLGFNPMDPTVEGKIGIWEDGAGT     175
WP_181487234.1              ATEAGYANQCIYNSINVAAEDEEIENLTNSDVEASIVLCFDPMDPSVGGKLNVLNDGGKT     175
WP_010870368.1              ATEAGYAKQCIYNSINVSIDEQEYQVLVESDLEASIVLCFDPMDPTVEGKINVLTNGGKT     175
WP_018153670.1              ATEAGYASQCIYNSINVSIEEAEFQNLVESDLEASIVLCFDPMDPSVEGKLNVLLDGGKT     175
AEH06918.1                  ATEAGYADQCIYNSINVSMDDAEFQNLVDSDLEASIVLCFDPMDPSVQGKLNVLENGGKT     175
                              : *  .: ::**:* :    : : : .:. .::*** *:  * :* *:  :   *

WP_012618921.1              GQSKGMIQIAEEAGITRPILDTAATPLGLGSGGSYREILACKGIYGLPTGGAYHNMTVSW     236
QXO93990.1                  GQEKSMMAIAEECGIKRPILDTAATPLGLGSGGSFREILACKAIHGLPTGGAYHNMTVSW     236
CAA74773.1                  VSEKGMLEISDECGIENPLIDTAYTPFGSGAGTAYKVTLAVKAKLGLPVGGAPHNVPSAW     239
CAA10790.1                  -LTKGMLQISEECGIKYPLIDVAAMPLGAGSGPTIRSIPTMKAKFGLPIGGGYHNMASAW     235
WP_011033486.1              -QTKGMLQVAKECGIKYPIIDVAAMPLGAGSGATIRSVPTLKGKFGLPIGGGYHNMASAW     235
sp|Q8TU06.1|                -QTKGMLQVAEECGIKIPLIDVAAMPLGAGSGATIRSIPTIKGKLGLPVGGGYHNMASAW     235
WP_013296329.1              -IDKGLLEMAADCGIDKYLMDVAVTPLGQGAGVAVRTSFAVKSKWGYPVGSGIHNVPSAW     234
SCM58293.1                  -IDKGLLEMAADCGIDKYLMDVAVTPLGQGAGVAVRTSFAVKSKWGYPVGSGIHNVPSAW     234
WP_181487234.1              -KDIGMLELAEKAGIKYPLIDVAVTPMGNGAGHAVRAAFAVKAKLGLPVGSGIHNVPSAW     234
WP_010870368.1              -ADKGMLELAEKAGIKYPLIDTAVTPLGNGAGAAVRASFAVKALFGYPVGSGIHNIPSAW     234
WP_018153670.1              -ADTGMLELAEKAGIKYPLIDVAVTPLGNGAGPAVRASFAVKAKLGLPVGSGIHNIPSAW     234
AEH06918.1                  -ADTGMLELAEKAGIKYPLVDVAVTPLGNGAGPAVRAAFAEKATYGYPVGSGVHNIPSAW     234
                                .:: :: ..**   ::*.*  *:* *:* : :   : *.  * * *.. **:  :*
```

```
WP_012618921.1       TWLRRWKKN-LGTVYDGKPALKDQMLKHYAKDIEALKQAVWSAPDIGCNLIASTLGADLI     295
QXO93990.1           TWLKRWRKSGIIDRYKDAGTLLEQMGHHHFGGVEGIRQAAWSSADIGCNIMAATLGADLI     296
CAA74773.1           DWLRDFMKKL------------------KEEGKEEWAELAHESSDWGSNVVAATLCCDYL     281
CAA10790.1           DWLRKFKKTQ------------------PD------AKAIYMPADIGTNLVAQIAGSDYL     271
WP_011033486.1       DWLRKFKKTQ------------------PD------PKAIYMPTDIGTNLVAQIAGSDYL     271
sp|Q8TU06.1|         DWLRKFKKTQ------------------PD------PKAIYMPADIGTNLVAQIAGSDFL     271
WP_013296329.1       DWLREYKKEH---------------------------KEAWPVCDVGSNLIQQMAGGDFV     267
SCM58293.1           DWLRGYKKDH---------------------------KEAWPVCDVGSNLIQQMAGGDFV     267
WP_181487234.1       DWLREFRKGL------------------REEGKDQTAKDVHHVCDIGANIVQTMTSGDYV     276
WP_010870368.1       DWLREFRKQL------------------REAGEREKAKDIHHVCDVGANLVQVMASGDFV     276
WP_018153670.1       DWLREFRKGL------------------REEGLTQLAKDVHHVCDIGANIVQTMGAGDFV     276
AEH06918.1           DWLREFRKGL------------------REKGETQLSKDVHHVCDIGANIVQTMTSADFV     276
                      **: : *                             :      * * *::      * :

WP_012618921.1       MFGPIENMEPMLTTQAYADITILEAARDLGIDTQ-DPNHPIFKLI   339
QXO93990.1           MFGPIENCEATATAMAFSDIVLAETLRELGGDVK-AEKHPINMLV   340
CAA74773.1           LFGPIENAPAIWPVVAMVDALIVEANEDVGVEPQ-VEEHPANIVR   325
CAA10790.1           LYGPIENVNQIFPAVAMVDIMLGETAKDLGVEIADLENHPVTKLT   316
WP_011033486.1       LYGPIENVNQIFPAVAMVDIMLGETAKELGVEIADLENHPVTKLT   316
sp|Q8TU06.1|         LYGPIENVEKVFPAVAMVDIMLGETAKELGVEIADSENHPVTRLT   316
WP_013296329.1       LYGPIENARMAFPACAMADIFISEAAKDIGTEA--VEDHPFFKLL   310
SCM58293.1           LFGPIENARIAFPACAMADIFISEAAKDIGTEP--VEDHPFFKLL   310
WP_181487234.1       LYGPIDNAELAFPAVAMTDMIIAETAKEMGTMP--VAEHPLNKLI   319
WP_010870368.1       LYGPIDNAYMTFPAVAMVDAIIAEAAKELGIEP--IDTHPFKKLV   319
WP_018153670.1       LYGPIDNAQLAFPAVAMTDMVIAEAAKELGTAA--VDTHPFNKLL   319
AEH06918.1           LYGPIDNARLAFPAVAMTDAVIAEAAKEMGVTP--VDNHPINKLV   319
                     ::***:*      . *  *  : *: .::*        **   :
```

Fig. S7. Sequence alignment. Abbreviations used: M.marb, *Methanothermobacter marburgensis*; M.wolf, *Methanothermobacter wolfeii*, M.jann, *Methanocaldococcus jannaschii*; M.mari, *Methanococcus maripaludis*; M.bark, *Methanosarcina barkeri*; M.maze, *Methanosarcina mazei*; M.palu, *Methanosphaerula palustris*; M.hung, *Methanospirillum hungatei*; M.kand, *Methanopyrus kandleri*; M.okin, *Methanothermococcus okinawensis*; M.acet, *Methanosarcina acetivorans*; M.ther, *M. thermolithotrophicus* (A) MtrA. The significantly conserved linker between $MtrA_c$ and $MtrA_s$ is highlighted in green. Segments of MtrA close to the corrinoid ring and close to the B_{12} tail and those forming the interface to MtrH or MtrE (frequently overlapping) are shown in red, blue and orange, respectively. Residues forming the stalk interior are marked in magenta. (B) MtrB. Segment 7-15 (purple) forms an interface with MtrH according to Alphafold2. Invariant GlyB79 and GlyB87 (red) allow a close contact with MtrF. (C) MtrC, (D) MtrD, (E) MtrE. Regions contacting the $MtrA_s$/corrinoid ring, CoM and Na^+ are marked in red, green and magenta, respectively. Helices II and V constituting the channel are drawn in brown. (F) MtrF. Invariant residues GlyF36 and GlyF44, involved in helix kinking, are marked in blue. No MtrF was found in *M. maripaludis*. (G) MtrG. The segment forming the interior of the stalk (magenta) is significantly conserved. *M palustris and M. hungatei* do not contain MtrG. (H) MtrH. Residues in contact and in neighborhood to H_4MPT are shown in green. Residues close to $MtrA_s$/corrinoid are marked in red; those contacting or close to MtrB are marked in violet and blue, respectively.

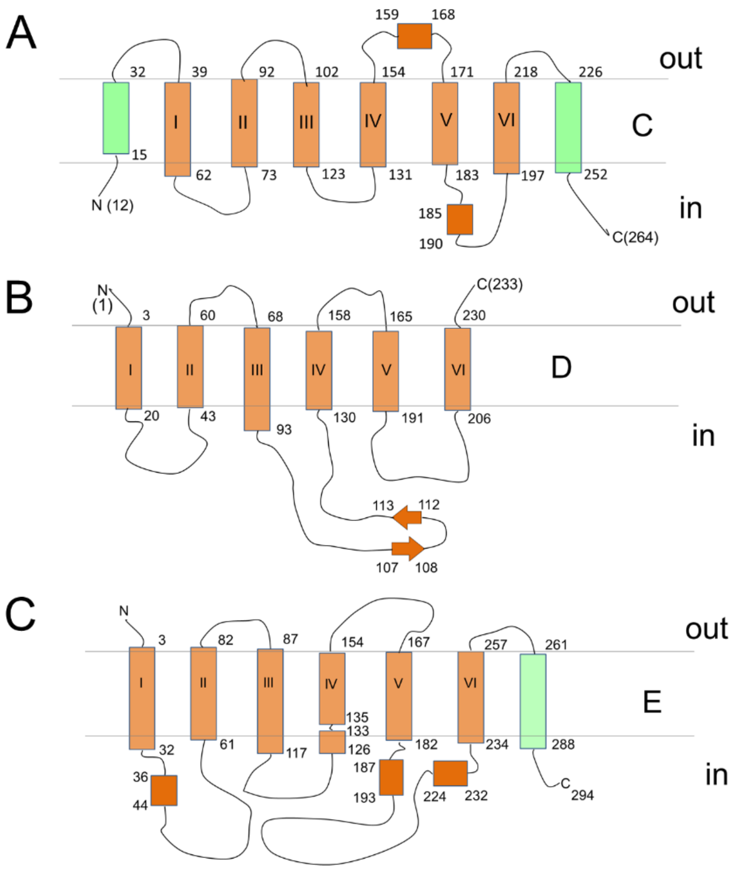

Fig. S8. Topology scheme of MtrC, MtrD and MtrE. Amino acids inside the membrane (documented by the two horizontal line) include segments C19-C35, C38-C55, C75-C92, C99-C119, C135-C157, C169-C183, C205-C220, C225-C239, D4-D21, D44-D60, D67-D84, D134-D154, D170-D187, D209-D226, E8-E25, E62-E79, E87-E103, E137-E155, E165-E181, E239-E257, and E260-E276 according to OREMPRO (21). (A) MtrC. MtrC contains, besides the six-helix bundle (orange), an N-terminal (14:33) and a C-terminal (226:252) transmembrane helix (green) involved in multi-subunit complex assembly. MtrC (and also MtrD and MtrE) are characterized by short extracellular loops except for helix C159-C168, which might be required for the release of Na^+ into the extracellular space. (B) MtrD. Besides its six-helix bundle MtrD is endowed with three expanded cytoplasmic segments after helices I (D3:D19), III (D65:D95), and V (D164:D192). They consist of several parallel irregular stretches partly joined to small β-sheet elements that build up one wall of the MtrCDE cavity and interact with B_{12}, MtrA and MtrE. (C) MtrE. The largest transmembrane subunit contains, besides the six-helix bundle, the segregated C-terminal anchor helix E258:E289 that forms an interface with the cytoplasmic segments following helices D65:D95 and E223:E232 and the MtrB linker B58-B73. MtrE contains a pronounced cytoplasmic subdomain with four functional important short helices. Helix Ia (E35:E44) is part of the Na^+ binding pocket and the MtrCDE cavity thereby involved in binding of CoM and the B_{12} nucleotide tail. Helix III (E86:E117) is prolonged into the cytoplasm and fixes helix Ia (E35:E44). Helices Va (E185:E194) and VIa (E223:E232) are involved in Na^+ binding (Fig. 4C).

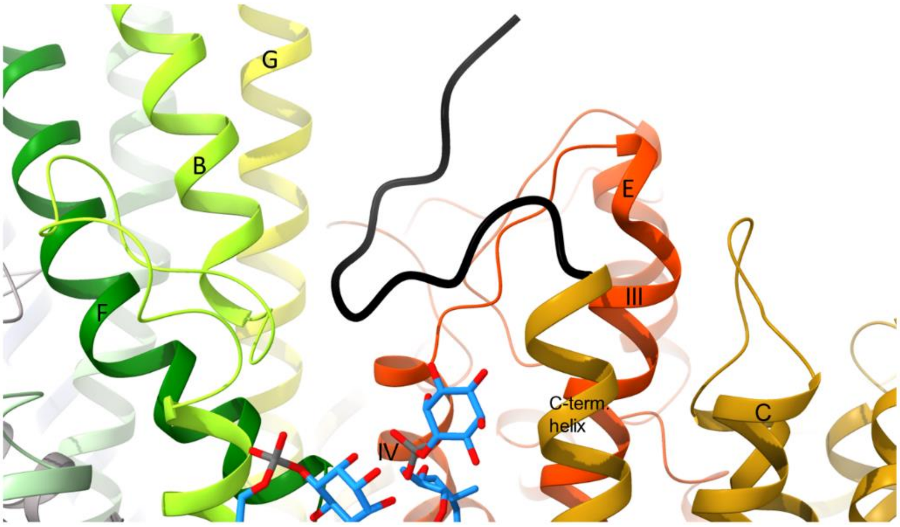

Fig. S9. Contact area between the C-terminal region of MtrC and the residual protein complex. The C-terminal helix (226:252) of MtrC (orange) is strongly associated with helix IV (131:154) and the preceding loop of MtrE. The following cytoplasmic C-terminal arm (black) of MtrC interacts, in addition, with MtrB, MtrF and MtrG of the stalk. As described (Fig. S6*C*), the phosphoinositol heads of tetraether glycolipids (carbon in blue) also contribute to the interaction network between the subunits.

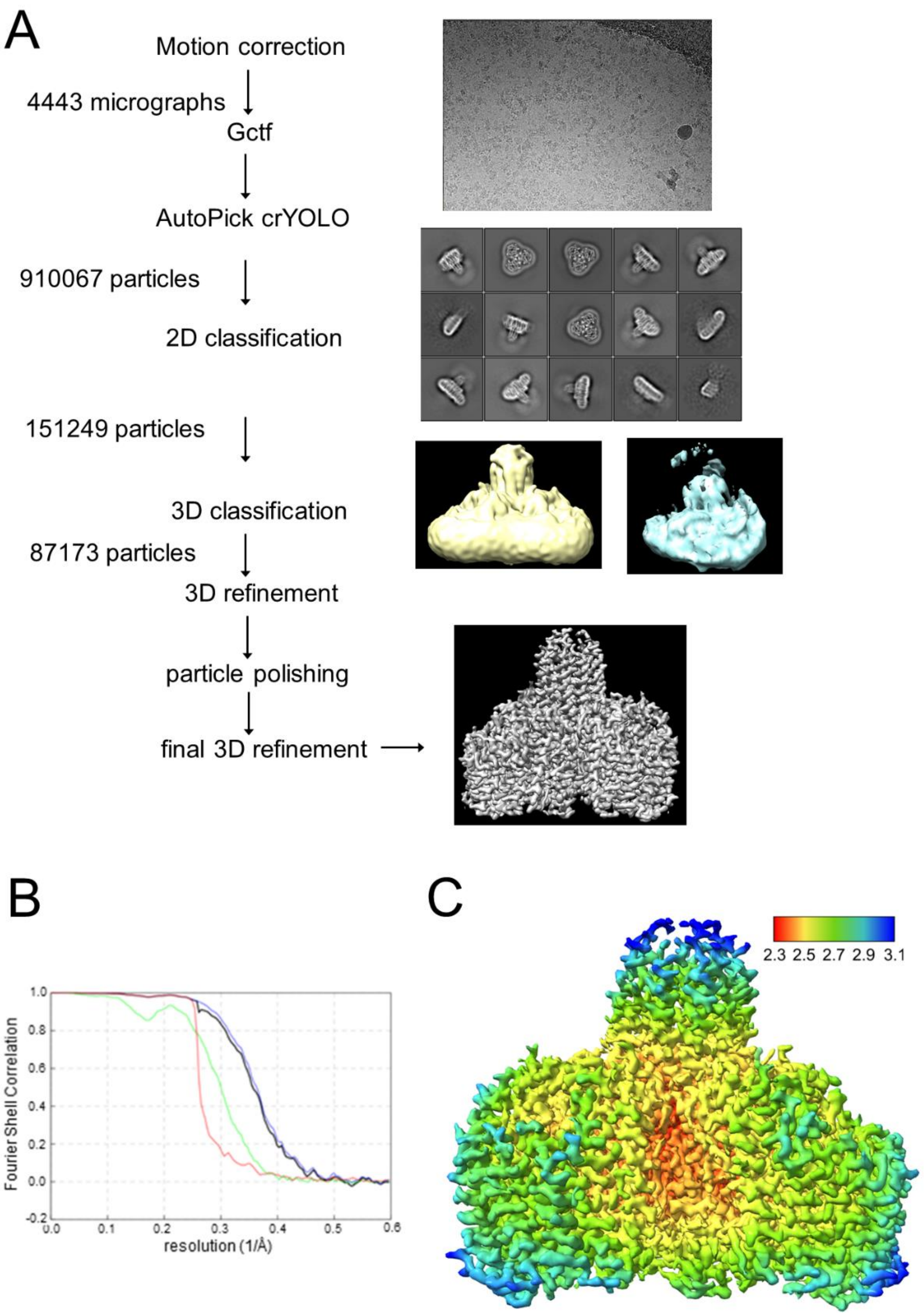

Fig. S10. Cryo-EM Mtr(ABCDEFG)$_3$-CoM structure determination using Relion. (A) Workflow of data processing including one image of a micrograph as well as 2D/3D classification and 3D refinement maps. (B) Gold-standard FSC plot (green curve: unmasked; blue: masked; red, phase randomized masked; black, FSC corrected for overfitting). Resolution estimated at FSC=0.143. (C) Map of the protein complex viewed parallel to the membrane plane colored by local resolution.

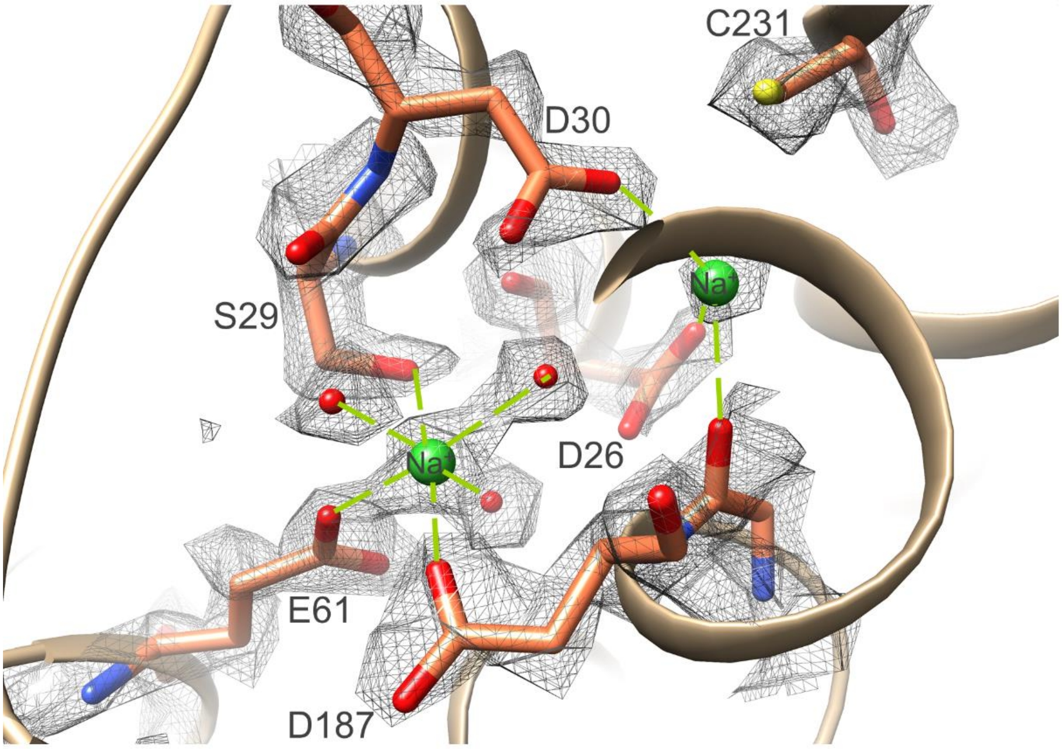

Fig. S11. Density modified map at the hydrophilic Na^{+} binding pocket. One metal binding site could be clearly detected according to the high quality of the density at 1.99 Å resolution and metal-ligand distances. The second potential metal binding site is only weakly occupied and cannot be definitively discriminated from a binding site of a firmly bound water molecule. A further attractive hydrophilic pocket lies adjacent to the conserved GluE28, GlnE34 and LysE56 but density for a metal was not visible. A further metal binding site was tentatively identified at the extracellular boundary between AlaB97-O, LeuB100-OXT and LeuA'235-O and three protein-linked solvent molecules involved in the fixation of different subunits. A Zn^{2+} binding site present in other thiol-activating methyltransferases (22, 23) or even nearby positioned potential ligands as cysteine, histidine and glutamate/aspartate were not found adjacent to CoM, although space is available in the oversized cavity.

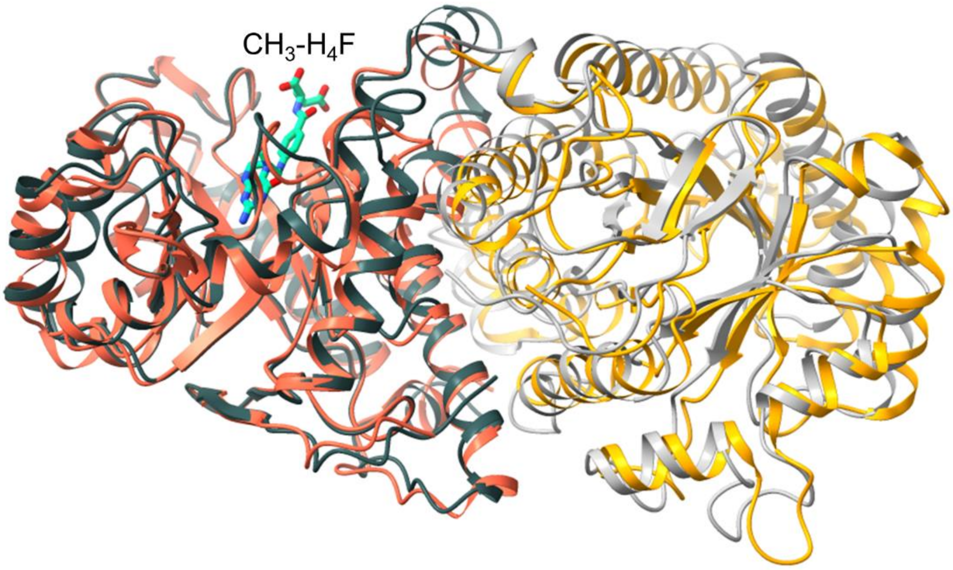

Fig. S12. Alphafold2 model of the MtrH dimer (dark-gray, light-gray) of *M. marburgensis*. The Alphafold2 structures of MtrH of *M. marburgensis*, *M. wolfeii*, *M. kandleri*, *M. barkeri*, *M. jannaschii* and *M. maripaludis* are virtually identical. The MtrH monomer reveals the well-known TIM barrel architecture that notably deviates from the classical fold by two antiparallel β-strands attached to the first TIM barrel strand covering the bottom of the TIM barrel and two helices downstream the prolonged helix H247:H262 involved in oligomerization. The MtrH dimer is superimposed with the MtgA dimer (tomato, orange) of *D. hafniense* determined in complex with methyl-H_4F (carbon in turquoise) (24). MtrH and MtgA rms deviate 1.9 Å (301 from 304 residues; 35% sequence identity; TM: 0.91). The high conservation of their monomer-monomer interfaces strongly argues for a homodimeric MtrH. Experimental hints about a homodimeric state of MtrH were obtained, when purifying MtrH of *M. marburgensis* from the membrane protein fraction after separating MtrH from the MtrABCDEFGH complex with DMMA (11). MtgA is involved in the N-demethylation of the quaternary amine glycine betaine, which is used for energy production and carbon assimilation in acetogenic bacteria. It catalyzes the methyl transfer from methyl-cob(III)alamin to tetrahydrofolate. After transfer of methyl-H_4F from the superimposed MtgA into MtrH an exchange of methyl-H_4F by methyl-H_4MPT is feasible without noticeable interference with the polypeptide. The related H_4MPT/H_4F binding mode is corroborated by the found methylation reaction of methyl-H_4F by MtrABCDEFGH of *M. mazei (25).* The structurally related methyltransferase H_4F dependent AscE of *Moorella thermoacetica* catalyzes the methyl transfer from methyl-H_4F to the corrinoid iron-sulfur protein (26) and to the H_4F binding domain of methionine synthase from *E.coli* (27). The rms deviation between MtrH and AscE is 3.3 Å (242 of 258 residues; TM: 0.71) (28).

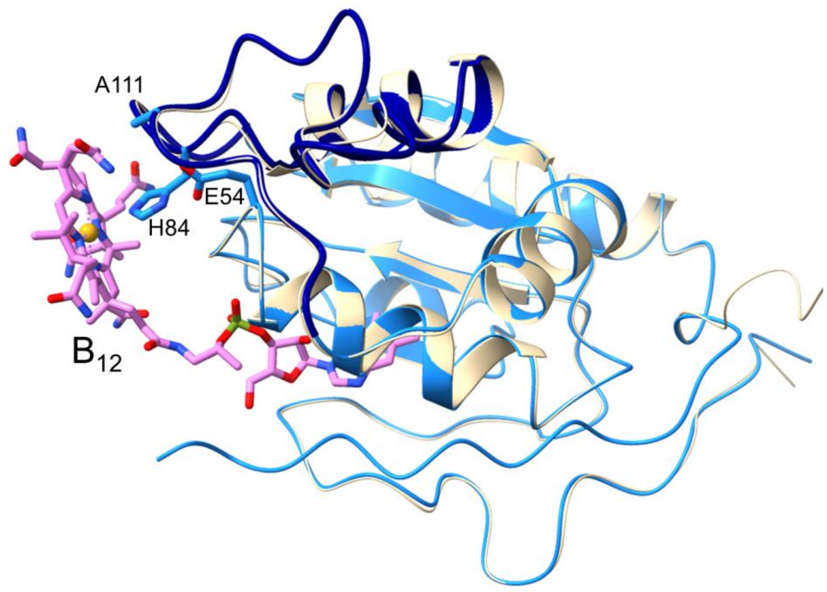

Fig. S13. Alphafold2 model of MtrA from *M. marburgensis*. MtrA (blue) adopts a Rossmann fold and is nearly identical with the MtrA homolog of *M. fervidus* (tan) (29) reflected in a rms deviation of 0.49 Å (160 of 163 residues; TM: 0.82). B_{12} (pink) was transferred into *M. marburgensis* $MtrA_s$ with minor side chain adjustments to avoid a collision. The exposed corrinoid interacts with loops after strands A29:A31, A47:A50 and A73:A77 as well as segment A111-A112. As described previously, the binding mode of B_{12} is rather different in MtrA compared to most other B_{12}-containing proteins (29) despite the common Rossmann fold scaffold (30). HisA84 was identified as the Co ligand by site-directed mutagenesis (31) and confirmed by structural data (29). The segment A80-A115 (dark-blue) including helix A85:A94 is perhaps strongly influenced by the position of HisA84, which in turn depends on whether Co is in the square-planar Co(I) and the octahedral CH_3-Co(III)(His-on) states. HisA84-NE1 is hydrogen-bonded with GluA54-OE1; the latter interacts with GluA54N and AlaA111N.

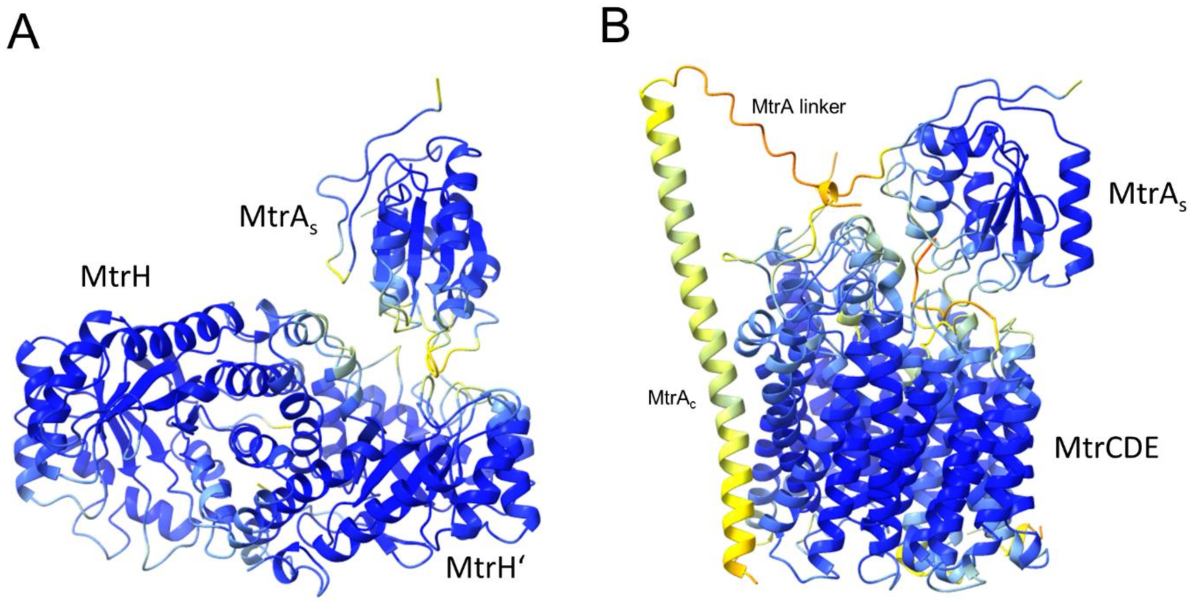

Fig. S14. Quality analysis of Alphafold2 calculations (32) of the $MtrA_s$-MtrH and MtrA-MtrCDE models. (A) pLDDT (predicted local distance difference test) coloring (blue to yellow) representation of the $MtrA_s$-MtrH subcomplex from *M. marburgensis*. The pLDDT values of the MtrH dimer and $MtrA_s$ are very high and the prediction reliable, but those of the contact region are relatively low (orange-green) and cannot be applied as single information source. However, a similar interface was obtained by independent calculations for the *M. marburgensis*, *M. thermoautotrophicum*, *M. wolfeii*, *M. mazei*, *M. jannaschii* and *M. hungatei* $MtrA_s$-MtrH subcomplexes. Moreover, the evaluation of these results has to consider that the exposed corrinoid of $MtrA_s$ (but also methyl-H_4MPT of MtrH) is not included in the Alphafold2 calculations. However, the corrinoid forms a significant fraction of the contact area and also influences the conformation of surrounding segments of $MtrA_s$ that also partly form the interface to MtrH (Fig. 5A). (B) pLDDT coloring (blue to yellow) representation of the MtrA-MtrCDE subcomplex. The pLDDT values of residues of the interface region are rather poor. Again, B_{12} is a major component of the contact area (Fig. 5B). Proteins from M. *marburgensis, M. wolfeii, M. jannaschii, M. kandleri, M. barkeri* and *M. maripaludis* revealed a similar interface. The conformation of the MtrA linker is highly undefined according to Alphafold2 calculations, which reflects its function. The position of the C-terminal MtrA helix $MtrA_c$ cannot be assessed, as the other stalk helices were not included in the calculation.

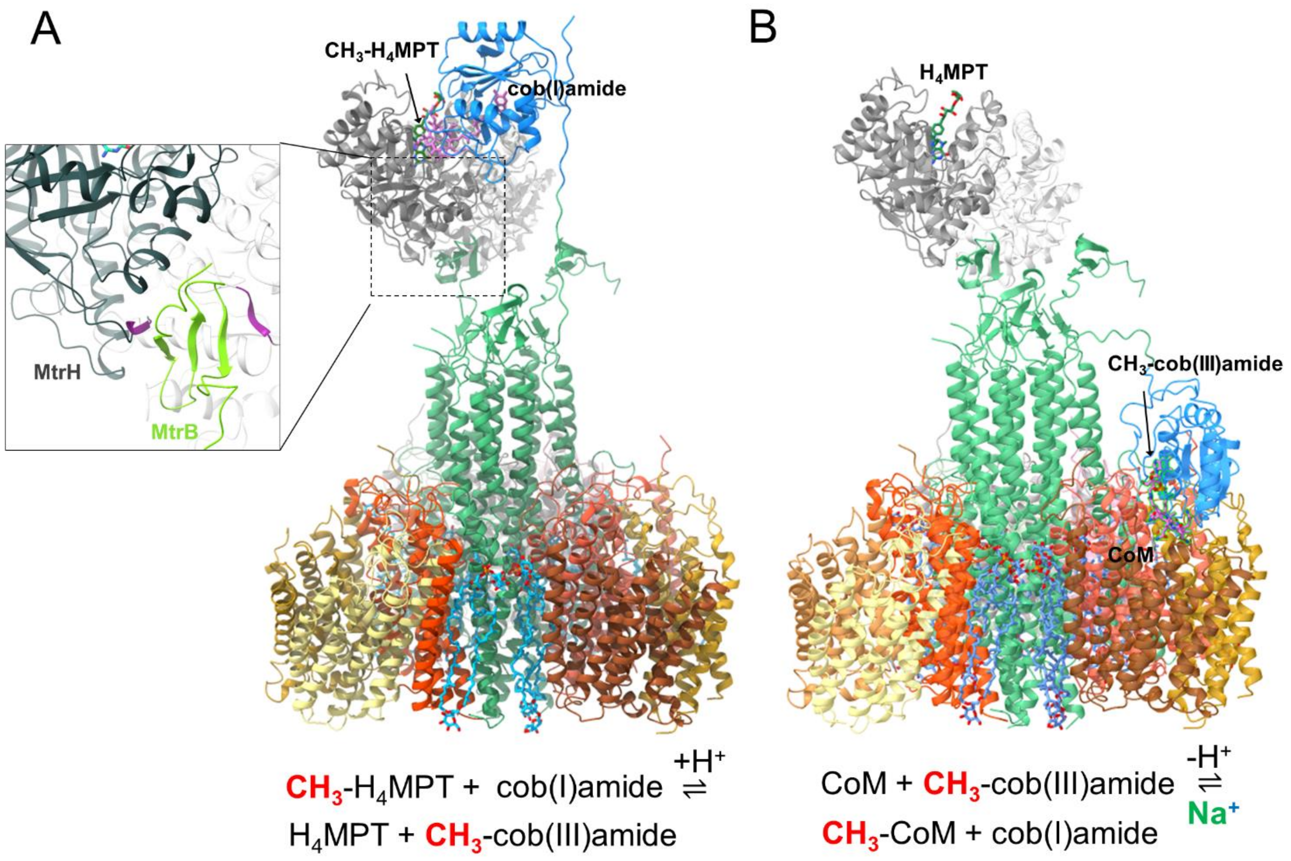

Fig. S15. Cartoon representation of the Mtr(ABCDEFG)$_3$H$_2$ complex in the conformation of the two half-reactions. (A) The methyl-H$_4$MPT demethylation state. The model is composed of the cryo-EM Mtr(A$_c$BCDEFG)$_3$ complex, the MtrBH$_2$ module from the superimposed Alphafold2 model Mtr(A$_c$BFG)$_3$H$_2$ (MtrA$_c$BFG green, MtrH monomers dark-gray and bright-gray) and MtrA$_s$ (blue) of the superimposed MtrA$_s$H$_2$ model (Fig. 5A). The visible subunits are underlined. MtrH$_2$ predominantly forms an interface with MtrB but MtrF and MtrG might also contribute. Alphafold2 models of the MtrB-MtrH$_2$ subcomplex calculated from *M. marburgensis*, *M. wolfeii*, *M. jannaschii*, *M. kandleri*, *M. barkeri* and *M. maripaludis* sequences are virtually identical. As presented in the inset, the N-terminal 25 amino acids of MtrB, which are disordered in the cryo-EM map fold as a flat three-stranded antiparallel β-sheet (green) localized at the top of the stalk. The three β-strands are placed into the cleft formed between the two MtrH subunits such that the N-terminal residues H3-H5 of the latter add one strand each (magenta) to constitute a five-stranded mixed β-sheet. Moreover, an extended hydrophobic patch is formed between ValB7, IleB9, LeuB17, IleB24, PheH4, PheH213, PheH2, PheH'2 (the prime describes the second monomer of MtrH$_2$), PheH4' and PheH'213. LysB8 and AspH50 are linked by a salt bridge. In the presented Mtr(ABCDEFG)$_3$H$_2$ model the MtrA linker is rather strained but it is conceivable that MtrB-MtrH$_2$ might be more inclined towards the membrane thereby shortening the distance to the stalk and relaxing the MtrA linker. Residues AlaB27-GlyB29 belong neither to the three-stranded N-terminal β-sheet nor to the MtrB part of the stalk visible in the cryo-EM map and may act as a flexible linker. If this holds true, the three-stranded N-terminal β-sheet of MtrB (B1-B26) and the associated MtrH$_2$ are mobile. Thus, MtrH might be part of the vitrified protein complex used for cryo-

EM measurements but disordered in the density. Nevertheless, the current data cannot finally exclude that MtrH is only temporally bound to the MtrABCDEFG complex. The association of the $MtrH_2$ dimer violates the threefold symmetry of the residual $Mtr(ABCDEFG)_3$ complex. A homotrimeric MtrH model attached to the top of the stalk in contact with three MtrB N-terminal β-sheets could be generated by Alphafold2 calculations but only with a low probability. This hypothesis was therefore discarded, although a threefold symmetry of the entire $Mtr(ABCDEFGH)_3$ complex is intuitively attractive. No experimental data concerning the stoichiometry of MtrABCDEG and MtrH exist. (B) The CoM methylation state. The model is composed of the cryo-EM $Mtr(\underline{A_c}B\underline{CDEFG})_3$ structure, the $Mtr\underline{BH_2}$ module (see inset of a) from the superimposed Alphafold2 model $Mtr(A_cBFG)_3H_2$ and $Mtr\underline{A_s}$ of the superimposed Alphafold2 MtrA-MtrCDE model (Fig. 5B). Superposition between the experimental MtrCDE and Alphafold2 MtrA-MtrCDE results in a rmsd of 0.63 Å (772 of 795 amino acids). Only, a few side chains have to be adjusted in the calculated MtrCDE structure after modelling B_{12} except for the N-terminal arm of MtrC. If the hypothesis is valid that $MtrH_2$ is associated with MtrB, $MtrA_s$ hosting methyl-cob(III) (blue) swings from the top of the stalk towards the MtrCDE cavity next to the membrane over more than 100 Å.

References

1. R. K. Thauer, Biochemistry of methanogenesis: a tribute to Marjory Stephenson. 1998 Marjory Stephenson Prize Lecture. *Microbiology (Reading)* **144**, 2377-2406 (1998).
2. V. Müller (2008) Bacterial fermentation. in *Encyclopedia of Life Sciences* (John Wiley & Sons, Ltd, Chichester, United Kingdom), pp 251-281.
3. R. K. Thauer, The Wolfe cycle comes full circle. *Proc Natl Acad Sci U S A* **109**, 15084-15085 (2012).
4. S. Shima, G. F. Huang, T. Wagner, U. Ermler, Structural Basis of Hydrogenotrophic Methanogenesis. *Annual Review of Microbiology, Vol 74, 2020* **74**, 713-733 (2020).
5. A. K. Kaster, J. Moll, K. Parey, R. K. Thauer, Coupling of ferredoxin and heterodisulfide reduction via electron bifurcation in hydrogenotrophic methanogenic archaea. *Proc Natl Acad Sci U S A* **108**, 2981-2986 (2011).
6. R. K. Thauer, A. K. Kaster, H. Seedorf, W. Buckel, R. Hedderich, Methanogenic archaea: ecologically relevant differences in energy conservation. *Nat Rev Microbiol* **6**, 579-591 (2008).
7. W. Buckel, R. K. Thauer, Energy conservation via electron bifurcating ferredoxin reduction and proton/Na(+) translocating ferredoxin oxidation. *Biochim Biophys Acta* **1827**, 94-113 (2013).
8. C. Welte, U. Deppenmeier, Bioenergetics and anaerobic respiratory chains of aceticlastic methanogens. *Biochim Biophys Acta* **1837**, 1130-1147 (2014).
9. J. M. Kurth *et al.*, Methanogenic archaea use a bacteria-like methyltransferase system to demethoxylate aromatic compounds. *ISME J* **15**, 3549-3565 (2021).
10. K. Lang, J. Schuldes, A. Klingl, A. Poehlein, R. Daniel, A. Brune, New Mode of Energy Metabolism in the Seventh Order of Methanogens as Revealed by Comparative Genome Analysis of "Candidatus Methanoplasma termitum". *Applied and Environmental Microbiology* **81**, 1338-1352 (2015).
11. V. Upadhyay *et al.*, Molecular characterization of methanogenic N(5)-methyl-tetrahydromethanopterin: Coenzyme M methyltransferase. *Biochim Biophys Acta* **1858**, 2140-2144 (2016).
12. S. H. W. Scheres, A Bayesian View on Cryo-EM Structure Determination. *Journal of Molecular Biology* **415**, 406-418 (2012).
13. H. Morii, Y. Koga, Asymmetrical topology of diether- and tetraether-type polar lipids in membranes of Methanobacterium thermoautotrophicum cells. *J Biol Chem* **269**, 10492-10497 (1994).
14. O. Grather, D. Arigoni, Detection of Regioisomeric Macrocyclic Tetraethers in the Lipids of Methanobacterium-Thermoautotrophicum and Other Archaeal Organisms. *Journal of the Chemical Society-Chemical Communications* DOI 10.1039/c39950000405, 405-406 (1995).
15. L. M. F. Baumann *et al.*, Quantitative Analysis of Core Lipid Production in Methanothermobacter marburgensis at Different Scales. *Bioengineering (Basel)* **9**, 169 (2022).
16. C. Knappy *et al.*, Mono-, di- and trimethylated homologues of isoprenoid tetraether lipid cores in archaea and environmental samples: mass spectrometric identification and significance. *J Mass Spectrom* **50**, 1420-1432 (2015).
17. M. Y. Yoshinaga *et al.*, Methanothermobacter thermautotrophicus modulates its membrane lipids in response to hydrogen and nutrient availability. *Front Microbiol* **6**, 5 (2015).
18. Z. Han *et al.*, Structural insights into a spindle-shaped archaeal virus with a sevenfold symmetrical tail. *Proc Natl Acad Sci U S A* **119**, e2119439119 (2022).
19. C. T. Lloyd *et al.*, Discovery, structure and mechanism of a tetraether lipid synthase. *Nature* **609**, 197-203 (2022).
20. G. von Heijne, Membrane protein structure prediction. Hydrophobicity analysis and the positive-inside rule. *J Mol Biol* **225**, 487-494 (1992).
21. G. Postic, Y. Ghouzam, J. C. Gelly, OREMPRO web server: orientation and assessment of atomistic and coarse-grained structures of membrane proteins. *Bioinformatics* **32**, 2548-2550 (2016).
22. A. Hoeppner *et al.*, Structure of the corrinoid:coenzyme M methyltransferase MtaA from Methanosarcina mazei. *Acta Crystallogr D Biol Crystallogr* **68**, 1549-1557 (2012).

23. R. G. Matthews, C. W. Goulding, Enzyme-catalyzed methyl transfers to thiols: the role of zinc. *Curr Opin Chem Biol* **1**, 332-339 (1997).
24. T. Badmann, M. Groll, Structures in Tetrahydrofolate Methylation in Desulfitobacterial Glycine Betaine Metabolism at Atomic Resolution. *Chembiochem* **21**, 776-779 (2020).
25. T. Lienard, B. Becher, M. Marschall, S. Bowien, G. Gottschalk, Sodium ion translocation by N5-methyltetrahydromethanopterin: coenzyme M methyltransferase from Methanosarcina mazei Go1 reconstituted in ether lipid liposomes. *Eur J Biochem* **239**, 857-864 (1996).
26. D. L. Roberts, S. Zhao, T. Doukov, S. W. Ragsdale, The reductive acetyl coenzyme A pathway: sequence and heterologous expression of active methyltetrahydrofolate:corrinoid/iron-sulfur protein methyltransferase from Clostridium thermoaceticum. *J Bacteriol* **176**, 6127-6130 (1994).
27. R. V. Banerjee, N. L. Johnston, J. K. Sobeski, P. Datta, R. G. Matthews, Cloning and sequence analysis of the Escherichia coli metH gene encoding cobalamin-dependent methionine synthase and isolation of a tryptic fragment containing the cobalamin-binding domain. *J Biol Chem* **264**, 13888-13895 (1989).
28. T. Doukov, J. Seravalli, J. J. Stezowski, S. W. Ragsdale, Crystal structure of a methyltetrahydrofolate- and corrinoid-dependent methyltransferase. *Structure* **8**, 817-830 (2000).
29. T. Wagner, U. Ermler, S. Shima, MtrA of the sodium ion pumping methyltransferase binds cobalamin in a unique mode. *Sci Rep* **6**, 28226 (2016).
30. C. L. Drennan, S. Huang, J. T. Drummond, R. G. Matthews, M. L. Ludwig, How a protein binds B12: A 3.0 A X-ray structure of B12-binding domains of methionine synthase. *Science* **266**, 1669-1674 (1994).
31. U. Harms, R. K. Thauer, Identification of the active site histidine in the corrinoid protein MtrA of the energy-conserving methyltransferase complex from Methanobacterium thermoautotrophicum. *Eur J Biochem* **250**, 783-788 (1997).
32. J. Jumper *et al.*, Highly accurate protein structure prediction with AlphaFold. *Nature* **596**, 583-589 (2021).
